# Assessing the correlates of wasting among children under five and their mothers in the Bay and Hiran regions of Somalia

**DOI:** 10.7189/jogh.15.04308

**Published:** 2025-12-12

**Authors:** Samantha Grounds, Shelley Walton, Kemish Kenneth Alier, Sydney Garretson, Said Aden Mohamoud, Sadiq Mohamed Abdiqadir, Qundeel Khattak, Mohamud Ali Nur, Abdullahi Muse Mohamoud, Meftuh Omer Ismail, Mohamed Billow Mahat, Adam Abdulkadir Mohamed, Abdifatah Ahmed Mohamed, Marina Tripaldi, Nadia Akseer

**Affiliations:** 1 Johns Hopkins University, Department of International Health, Baltimore, USA; 2 Save the Children, Somalia Country Office, Mogadishu, Somalia; 3 Save the Children, Save the Children International, London, UK; 4 Federal Government of Somalia, Ministry of Health & Human Service, Mogadishu, Somalia

## Abstract

**Background:**

To address Somalia’s high wasting burden, it is imperative to understand the country’s context-specific correlates of wasting. We aimed to assess the correlates of wasting among children under five (CU5) and mothers in Bay and Hiran.

**Methods:**

We analysed midline (CU5 n = 956; mothers n = 1066) and endline (CU5 n = 833; mothers n = 1023) data from a randomised controlled trial. We explored child (weight-for-height z-scores (WHZ)) and maternal outcomes (mid-upper arm circumferences) via linear regression for children and via Poisson regression for children and mothers, implementing a backwards elimination approach in a hierarchical way. We stratified CU5 models by region and age.

**Results:**

CU5 wasting was 12.9% at midline and 14.4% at endline, with a higher burden in Hiran. At midline, maternal underweight and maternal open defecation adversely affected WHZ, while having >1 CU5 was protective. At endline, no maternal education, a high reduced coping strategy index, and not consuming animal-based protein were associated with lower WHZ, while maternal overweight was protective. Stratifying by age did not reveal additional correlates. The following additional correlates appeared when stratifying by region: child illness, household decision-making, and household head gender in Bay and household open defecation and non-food item kits in Hiran. Mothers’ wasting was 8% at midline and 12% at endline, with the following identified correlates: an unacceptable food consumption score, moderate-to-severe household hunger, and poor child stool disposal practices at midline and household open defecation at endline. Maternal decision-making was protective at midline.

**Conclusions:**

Our results highlight variation in the key correlates of wasting by region, season, and age and contribute to evidence on the multifactorial correlates of wasting, encouraging context-specific approaches addressing the immediate, underlying, and basic causes of malnutrition. The findings emphasise the importance of maternal nutrition for child nutrition and the need for interventions considering household food security, sanitation, and gender dynamics in this humanitarian setting.

**Registration:**

The cluster-RCT is registered at ClinicalTrials.gov (ID: NCT06642012).

The World Health Organization, United Nations Children’s Fund, and World Bank Group estimated that 45 million children under five (CU5) worldwide suffered from wasting in 2022, with close to 14 million CU5 experiencing severe wasting (Text S1 in the **Online Supplementary Document**) [1]. In 2022, the estimated global prevalence of wasting among CU5 was 6.8%, with an estimated 12.2 million CU5 in Africa affected by wasting, of whom nearly three million were severely wasted [1]. Wasting is not only a concern among young children, but also among pregnant and lactating women (PLW) [2,3]. Among the 12 countries, including Somalia, most severely impacted by the ongoing worldwide food and nutrition crisis, acute malnutrition among PLW increased by 25% from 2020 to 2022, totalling nearly seven million acutely malnourished PLW and adolescent females in 2022 [2,3].

Somalia’s 2020 Health and Demographic Survey estimated that 11.6% of CU5 in Somalia are wasted, with 6% severely wasted, and a recent publication estimated that 1.7 million CU5 in Somalia would suffer from acute malnutrition in 2024 [4–6]. Approximately 62.6% of CU5 in Bay and 59.6% of CU5 in Hiran were estimated to be acutely malnourished from January to December 2024 [6]. Within these regions, Baidoa and Belet Weyne districts show high overall moderate acute malnutrition and severe acute malnutrition median prevalences of 14.2% and 4.9% in Baidoa district and of 14.7% and 3.6% in Belet Weyne district, falling into the critical/phase four category of The Integrated Food Security Phase Classification’s acute malnutrition scale [7,8].

Despite Somalia’s high wasting burden, with child wasting being the primary determinant of disability-adjusted life years in the country, there is limited primary evidence around the correlates of wasting in Somalia [9]. Peer-reviewed primary research analysing household survey data from 2007–10 found sex, age, illness, vaccination, dietary and nutritional factors, maternal age and mid-upper arm circumference (MUAC), household size, number of CU5 in the household, environmental factors, and other demographic and social factors (livelihood, displacement, urbanisation, and conflict) to be associated with wasting among children 6–59 months of age in Somalia [10–14]. More recently, an analysis of the 2019 Somalia Micronutrient Survey identified household wealth and iron status as factors associated with wasting among CU5 [15]. No peer-reviewed primary research on the correlates of wasting among PLW or mothers of CU5 in Somalia was identified.

We seek to address this evidence gap and inform strategies to address wasting in Somalia by assessing the correlates of wasting among CU5 and their mothers in the Bay and Hiran regions, selected for their high acute malnutrition burdens [8,16]. Specifically, we aim to answer the following research question: What are the top correlates of wasting among CU5 and their mothers in the Bay and Hiran regions of Somalia?

Our specific objectives include:

1. Exploring the correlates of wasting among CU5 in the Bay and Hiran regions of Somalia, stratifying results by region and by child age (9–23 months *vs.* 24–59 months) and comparing by seasonality.
2. Exploring the correlates of wasting among mothers of CU5 in the Bay and Hiran regions of Somalia and comparing by seasonality.

## METHODS

### Data source & study setting

We used data from a cluster-randomised controlled trial (cRCT) to evaluate the effectiveness of a CashPlus for Nutrition program implemented by Save the Children (Text S2 in the **Online Supplementary Document**) [17,18]. We carried out this study in the Bay and Hiran regions in southwest Somalia, a humanitarian setting where flooding, droughts, and conflict have threatened livelihoods, increased food insecurity, and contributed to both displacement from these regions as well as arrivals of internally displaced persons to these regions throughout the years (Text S3 in the **Online Supplementary Document**) [19–25]. We followed the STROBE guidelines for reporting this analysis (Text S4 in the **Online Supplementary Document**) [26].

### Data collection

The cRCT collected baseline (May 2023), midline (September 2023), and endline (December 2023) data [17,18]. We used midline data, corresponding to the transition from the Xagaa dry season to the Deyr rainy season, and endline data corresponding to the Jilaal dry season [27]. In October 2023, between the midline and endline data collection points, major flooding occurred in Somalia, affecting an estimated 2.5 million people and displacing over a million [24,28,29]. We did not include baseline data because the main trial focused on wasting prevention; therefore, children were recruited only if they were not malnourished, and a low prevalence of wasting was expected in the baseline data [17]. However, baseline characteristics for the main trial are reported elsewhere, and additional analyses showed that children retained in the study sample at midline and endline had demographic and nutritional characteristics similar to those at baseline [18].

### Outcomes of interest

Our outcome of interest was wasting, defined by weight-for-height Z-scores (WHZ) for CU5 and by MUAC for mothers [30–35]. We calculated WHZ using the zscore06 command in Stata, defining child wasting by a WHZ<−2 standard deviations (SDs) from the median age- and sex-specific WHZ from the World Health Organization 2006 Child Growth Module [30–32,36–38]. We examined child wasting as a binary outcome at midline and endline with the overall CU5 samples via multivariable Poisson regression models. However, to examine the age- and region-specific correlates of child wasting and due to sample size limitations and the rarity of wasting outcomes when considering these stratified models, we examined WHZ as a continuous outcome via linear regression models to allow for a broader exploratory analysis of the factors correlated with child nutritional status. For the mothers’ models, we categorised MUAC as a binary outcome to examine wasting, defined as a MUAC<23 cm, via multivariable Poisson regression models [33–35].

### Covariates

We considered variables that had complete or <15% missing responses and that could plausibly be related to wasting outcomes. Additionally, to address covariate imbalance that may affect regression results, we did not assess variables that had limited variation in each analytical sample (using a threshold of >75%/<25%) in the corresponding model. We considered variables concerning location, wealth and expenditure, displacement, household decision-making and women’s empowerment, intervention components, household food security and environment, water, sanitation, and hygiene (WASH) practices, health- and nutrition-related knowledge and practices, child diet and disease, and maternal characteristics (Table S1 in the **Online Supplementary Document**). We provide variable calculations, definitions, and thresholds (Table S2 and Text S5 in the **Online Supplementary Document**). Complete data on household crowding were available only in the midline data, and displacement, head of household, and maternal education data were collected only at endline. Regarding intervention components, according to Save the Children Somalia country office criteria, WASH non-food item (NFI) kits were targeted to vulnerable households based on factors such as communities without adequate WASH markets, communities with new internally displaced persons or those not getting support, and communities with larger populations lacking adequate WASH facilities. Mother-to-mother (M2M) support groups were formed in seven villages in Hiran and four in Baidoa, with villages selected based on factors such as service integration capacity and the prevention of interference with related activities/interventions [17,18].

### Analysis methods

**Figure 1:**
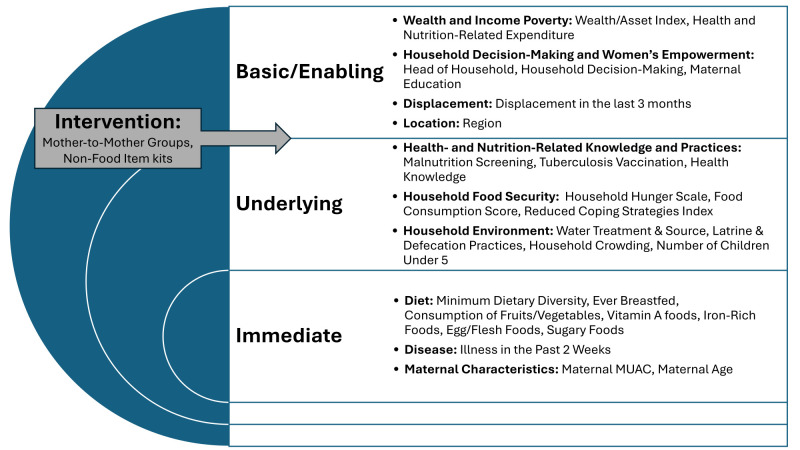
Mapping of variables tested in the CU5 models. CU5 – children under five.

We adapted the United Nations Children’s Fund Conceptual Framework on Maternal and Child Nutrition into a statistical framework for analysis, organising variables into the basic/enabling, underlying, and immediate levels of determinants of maternal and child nutrition (**Figure 1**; Figure S1 in the **Online Supplementary Document**) [39–41]. Additionally, we mapped intervention-related indicators into a separate level between the basic/enabling and underlying levels.

We analysed CU5 and mothers from the cRCT (Text S6 in the **Online Supplementary Document**). Details on the study population, trial participant selection criteria, and sample size calculation for the cRCT are provided elsewhere [17].

We separately and cross-sectionally analysed the midline and endline data, and implemented a backwards elimination model-building approach in a hierarchical way, as proposed by Victora and used in the literature [39–42]. We performed a complete case analysis. During the first stage of analysis, we examined unadjusted bivariate relationships between the outcome and each independent variable. Next, we examined major confounder-adjusted relationships, adjusting for child sex and age, household region, and trial arm in the CU5 models and adjusting for household region and trial arm in the mothers' models. From these major confounder-adjusted regression results, we retained any independent variable that had an association with the outcome with a *P*-value <0.20 for the final stage, as used elsewhere for studying determinants of maternal and child nutrition status [42]. We then used a backwards elimination model-building approach and implemented it in a hierarchical way at four levels according to the statistical framework [41]. We retained variables with *P*-values ≤0.10 in the final fully adjusted models. We adjusted for the cRCT trial arm; however, to not overfit the models, we did not control for the village cluster.

For examining wasting and WHZ among CU5, we built ‘overall’ models with the entire samples of children with both the midline data and endline data separately. We stratified additional models examining WHZ by region and child age (9–23 months *vs.* 24–59 months). For mothers’ wasting, we built only overall models with all mothers at midline and endline.

We assessed model fit checks for all final adjusted models (Text S7 in the **Online Supplementary Document**). We set the statistical significance level at α = 0.05 (*P* ≤ 0.05), with 0.05 < *P *≤ 0.10 considered marginally significant. We used Stata, version 18.5 (StataCorp, College Station, Texas, USA) for all analyses.

## RESULTS

### Participant characteristics

After applying the sample criteria, we retained 956 CU5 and 1066 mothers at midline, and 833 CU5 and 1023 mothers at endline, from the cRCT (**Table 1**; Figures S2–5 and Table S3 in the **Online Supplementary Document**).

**Table 1:** Key child, mother, and household characteristics*

	Midline, n (%)	Endline, n (%)
**Immediate level**		
Child sex	956	833
*Male*	473 (49.5)	424 (50.9)
*Female*	483 (50.5)	409 (49.1)
Child age, in months	956	833
*9–23*	260 (27.2)	222 (26.7)
*24–59*	696 (72.8)	611 (73.3)
Child illness in the past two weeks	956	833
*Yes*	308 (32.2)	253 (30.4)
*No*	648 (67.8)	580 (69.6)
Child ever breastfed	956	833
*Breastfed*	779 (81.5)	746 (89.6)
*Never breastfed*	177 (18.5)	87 (10.4)
Maternal age, in years	1066	1023
*<24*	164 (15.4)	157 (15.4)
*24–34*	486 (45.6)	464 (45.4)
*≥35*	416 (39.0)	402 (39.3)
**Underlying level**		
Household FCS	1066	1023
*Acceptable†*	718 (67.4)	542 (53.0)
*Unacceptable‡*	348 (32.7)	481 (47.0)
HHS	1066	1023
*Little-to-no hunger†*	790 (74.1)	545 (53.3)
*Moderate-to-severe hunger‡*	276 (25.9)	478 (46.7)
Household use of piped water source	1065	1023
*Piped*	216 (20.3)	352 (34.4)
*Other*	849 (79.7)	671 (65.6)
Household open defecation	1064	1023
*Yes*	253 (23.8)	261 (25.5)
*No*	811 (76.2)	762 (74.5)
**Basic/enabling level**		
Household region	1066	1023
*Bay*	452 (42.4)	460 (45.0)
*Hiran*	614 (57.6)	563 (55.0)
Household decision-making	1066	1023
*Joint*	610 (57.2)	673 (65.8)
*Maternal*	224 (21.0)	213 (20.8)
*Paternal*	232 (21.8)	137 (13.4)
Household wealth/asset index	1066	1023
*Not poor*	591 (55.4)	610 (59.6)
*Poor*	475 (44.6)	413 (40.4)

HHS – household hunger scale, FCS – food consumption score

*Child characteristics are according to child samples at midline and endline. Mother and household characteristics are according to the mother samples at midline and endline.

†Corresponding to Integrated Food Security Phase Classification 1/2 (*i.e.* minimal/stressed).

‡Corresponding to Integrated Food Security Phase Classification 3/4/5 (*i.e.* crisis/emergency/famine).

### Nutrition status

Average WHZ was −0.7 SD (95% confidence interval (CI) = −0.75, −0.61) at midline and −0.8 SD (95% CI = −0.89, −0.74) at endline. Approximately 13% (95% CI = 10.9, 15.1) of children were wasted at midline, and over 14% (95% CI = 12.2, 17.0) were wasted at endline. At both time points, the burden of wasting was higher in Hiran than in Bay and higher among older children than younger children, prompting stratified analyses by region and by age.

At midline, around 8% (95% CI = 6.7, 10.0) of mothers were wasted, whereas approximately 12% (95% CI = 10.1, 14.1) were wasted at endline. While maternal wasting was slightly higher in Hiran than in Bay, this difference was not statistically significant at midline (*P* = 0.182) or endline (*P* = 0.579) (**Table 2**).

**Table 2:** Nutrition status of children and mothers

			Region				Child age, in months			
	**Overall**		**Bay**		**Hiran**		**9–23**		**24–59**	
	**Midline**	**Endline**	**Midline**	**Endline**	**Midline**	**Endline**	**Midline**	**Endline**	**Midline**	**Endline**
**Children, n**	956	833	401	365	555	468	260	222	696	611
WHZ*	–0.7 (0.04; –0.75, –0.61)	–0.8 (0.04; –0.89, –0.74)	–0.3 (0.05; –0.38, –0.18)	–0.5 (0.05; –0.62, –0.40)	–1.0 (0.05; –1.07, –0.88)	–1.1 (0.05; –1.16, –0.96)	–0.4 (0.07; –0.51, –0.23)	–0.5 (0.08; –0.71, –0.39)	–0.8 (0.04; –0.88, –0.71)	–0.9 (0.04; –1.00, –0.83)
Wasting status†										
*Wasted*	123 (12.9; 10.89, 15.14)	120 (14.4; 12.18, 16.96)	16 (4.0; 2.46, 6.42)	26 (7.1; 4.89, 10.27)	107 (19.3; 16.20, 22.78)	94 (20.1; 16.69, 23.97)	20 (7.7; 5.01, 11.64)	24 (10.8; 7.34, 15.65)	103 (14.8; 12.35, 17.64)	96 (15.7; 13.03, 18.82)
*Not wasted*	833 (87.1; 84.86, 89.11)	713 (85.6; 83.04, 87.82)	385 (96.0; 93.58, 97.55)	339 (92.9; 89.73, 95.11)	448 (80.7; 77.22, 83.80)	374 (79.9; 76.03, 83.31)	240 (92.3; 88.36, 94.99)	198 (89.2; 84.35, 92.66)	593 (85.2; 82.36, 87.65)	515 (84.3; 81.18, 86.97)
**Mothers, n**	1066	1023	452	460	614	563				
MUAC, in cm*	27.49 (0.13; 27.24, 27.74)	27.27 (0.13; 27.01, 27.53)	27.49 (0.19; 27.12, 27.85)	27.17 (0.19; 26.79, 27.56)	27.49 (0.17; 27.15, 27.83)	27.35 (0.18; 26.99, 27.70)				
Wasting status, in cm†										
*Wasted (<23)*	87 (8.2; 6.66, 9.97)	122 (11.9; 10.08, 14.06)	31 (6.9; 4.86, 9.60)	52 (11.3; 8.71, 14.55)	56 (9.1; 7.08, 11.67)	70 (12.4; 9.95, 15.43)				
*Within range (23–30)*	730 (68.5; 65.62, 71.20)	673 (65.8; 62.82, 68.64)	312 (69.0; 64.61, 73.13)	309 (67.2; 62.74, 71.32)	418 (68.1; 64.28, 71.65)	364 (64.7; 60.60, 68.50)				
*Overweight (>30)*	249 (23.4; 20.91, 26.00)	228 (22.3; 19.84, 24.94)	109 (24.1; 20.38, 28.29)	99(21.5; 17.99, 25.52)	140 (22.8; 19.65, 26.30)	129 (22.9; 19.62, 26.58)				

MUAC – mid-upper arm circumference, WHZ – weight-for-height z-score, x̄ – mean

*Variables presented as mean (standard error; 95% confidence interval).

†Variables presented as count (proportion; 95% confidence interval).

### Correlates of CU5 wasting

**Figure 2:**
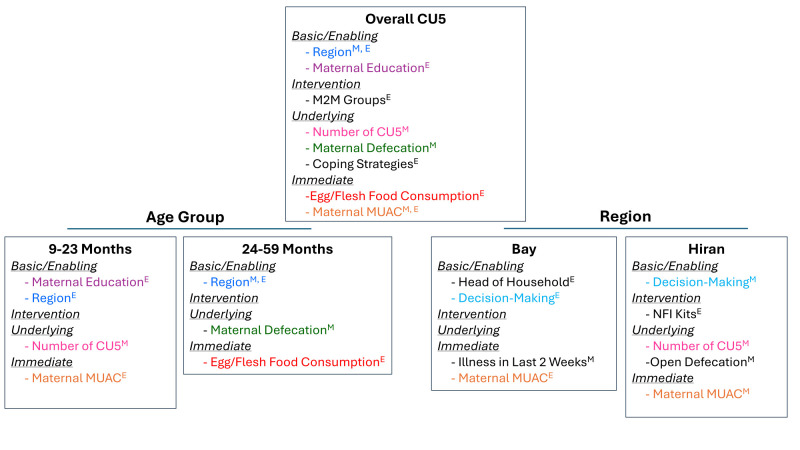
Correlates of CU5 WHZ in the final adjusted models. CU5 – children under five, WHZ – weight-for-height Z-score. Colour coding indicates that a variable was a statistically significant correlate of child WHZ in multiple CU5 models. ‘M’ superscript indicates the variable was a statistically significant correlate in the given model at midline, ‘E’ superscript indicates the variable was a statistically significant correlate in the given model at endline, and ‘M,E’ indicates the variable was a statistically significant correlate in both the midline and endline final adjusted models. This image depicts statistically significant correlates from the final adjusted models at the α = 0.05 level; additional correlates may have been marginally statistically significant or statistically significant in the unadjusted or basic confounder-adjusted models.

The presented results are the statistically significant findings from the final multivariable linear regression models with the hierarchical approach for child WHZ (**Figure 2**). Additional indicators were marginally statistically significant or were statistically significant in the crude or basic-adjusted models, and these results are available in the data tables. Relevant findings from the multivariable Poisson regression models assessing wasting as a binary outcome are highlighted here (Tables S4 and S5 in the **Online Supplementary Document**).

At the basic/enabling level, household region was a statistically significant correlate of WHZ at both midline and endline in the fully adjusted overall models. Children in Hiran had an average WHZ −0.66 SD (95% CI = −0.80, −0.52) and −0.58 SD (95% CI = −0.74, −0.43) lower than children in Bay at midline and endline, respectively, demonstrating a large effect size. These findings are consistent with results from the multivariable Poisson regression models, where, at both midline and endline, children living in Hiran *vs.* Bay had an increased relative risk of wasting (Tables S4 and S5 in the **Online Supplementary Document**). The education status of mothers was statistically significantly associated with WHZ at endline, although at a relatively small effect size, with having no education (compared to some education) corresponding to a −0.17 SD (95% CI = −0.33, −0.02) lower WHZ in the final overall adjusted model (**Table 3**).

**Table 3:** Correlates of CU5 WHZ at midline and endline

	Midline data results (n = 930)*						Endline data results (n = 833)*					
	**Unadjusted bivariate linear regression**		**Major confounder-adjusted linear regression†**		**Final multivariable linear regression (hierarchical approach)†**		**Unadjusted bivariate linear regression**		**Major confounder-adjusted linear regression†**		**Final multivariable linear regression (hierarchical approach)†**	
	***β* (95% CI)**	***P-*value**	***β* (95% CI)**	***P-*value**	***β* (95% CI)**	***P-*value**	***β* (95% CI)**	***P-*value**	***β* (95% CI)**	***P-*value**	***β* (95% CI)**	***P-*value**
**Basic/enabling level**												
Wealth/asset index (ref: not poor)												
*Poor*	0.34 (0.19, 0.48)	0.000	–0.00 (–0.16, 0.16)	0.964			0.38 (0.22, 0.53)	0.000	–0.10 (–0.31, 0.11)	0.360		
Health and nutrition-related expenditure (ref: middle 50%)												
*Lower 25%*	0.09 (–0.09, 0.27)	0.307	–0.00 (–0.17, 0.17)	0.992			–0.05 (−0.24, 0.13)	0.585	–0.11 (–0.29, 0.07)	0.222	–0.08 (–0.25, 0.09)	0.366
*Upper 25%*	–0.09 (–0.27, 0.09)	0.310	0.06 (–0.11, 0.23)	0.468			0.03 (–0.15, 0.22)	0.717	0.15 (–0.03, 0.33)	0.111	0.16 (–0.03, 0.34)	0.096
Head of household (ref: mother)												
*Father or other*	NA		NA		NA		0.10 (–0.05, 0.26)	0.186	–0.07 (–0.22, 0.08)	0.372		
Decision-making around income, purchases, & health care (ref: joint)												
*Maternal*	–0.25 (–0.43, –0.07)	0.008	–0.08 (–0.25, 0.09)	0.365			–0.25 (–0.44, –0.07)	0.007	–0.11 (–0.29, 0.07)	0.229		
*Paternal*	–0.31 (−0.49, −0.13)	0.001	–0.03 (–0.21, 0.15)	0.749			–0.20 (–0.43, 0.02)	0.079	–0.05 (–0.27, 0.16)	0.625		
Maternal education (ref: some education)												
*No education*	NA		NA		NA		–0.10 (−0.26, 0.07)	0.255	–0.19 (–0.34, –0.03)	0.021	–0.17 (–0.33, –0.02)	0.030
Region (ref: Bay)												
*Hiran*	–0.69 (–0.84, –0.55)	0.000	–0.66 (–0.80, –0.52)	0.000	–0.66 (–0.80, –0.52)	0.000	–0.55 (–0.70, –0.40)	0.000	–0.53 (–0.68, –0.38)	0.000	–0.58 (–0.74, –0.43)	0.000
**Intervention level**												
Attending M2M groups (ref: attended)												
*Did not attend*	–0.16 (–0.30, –0.01)	0.035	0.08 (–0.07, 0.22)	0.307			0.12 (–0.04, 0.29)	0.148	0.18 (0.01, 0.35)	0.037	0.17 (0.01, 0.33)	0.043§
NFI Kits (ref: received)												
	NA		NA		NA		0.16 (0.00, 0.31)	0.045	–0.03 (–0.18, 0.13)	0.732		
**Underlying level**												
HHS (ref: little-to-no hunger‡)												
*Moderate-to-severe hunger*§	–0.34 (–0.51, –0.18)	0.000	–0.02 (–0.19, 0.14)	0.787			–0.16 (–0.32, –0.01)	0.034	0.05 (–0.11, 0.20)	0.551		
FCS (ref: acceptable‡)												
*Unacceptable*§	–0.12 (–0.27, 0.04)	0.147	–0.04 (–0.19, 0.10)	0.553			–0.20 (–0.35, –0.05)	0.009	–0.12 (–0.26, 0.03)	0.124		
rCSI (ref: <19‡)												
*≥19*§	NA		NA		NA		–0.20 (–0.36, –0.03)	0.018	–0.14 (–0.29, 0.02)	0.093	–0.17 (–0.33, –0.01)	0.039
Household crowding (ref: not crowded)												
*Crowded*	–0.42 (–0.56, –0.27)	0.000	–0.00 (–0.18, 0.17)	0.965			NA		NA		NA	
Number of CU5 (ref: 1 child)												
≥*2 children*	0.28 (0.12, 0.44)	0.001	0.27 (0.10, 0.43)	0.002	0.24 (0.07, 0.40)	0.006	NA		NA		NA	
Water source (ref: piped water)												
*Not piped*	NA		NA		NA		–0.08 (–0.24, 0.08)	0.327	0.03 (–0.12, 0.19)	0.659		
Household defecation (ref: latrine/toilet)												
*Open area*	NA		NA		NA		–0.09 (–0.27, 0.08)	0.296	–0.02 (–0.19, 0.15)	0.846		
Child stool disposal (ref: latrine/toilet)												
*Open area*	–0.21 (–0.36, –0.06)	0.006	–0.21 (–0.36, –0.07)	0.004			–0.18 (–0.33, –0.02)	0.024	–0.13 (–0.27, 0.02)	0.094		
Maternal defecation (ref: latrine/toilet)												
*Open area*	–0.47 (–0.63, –0.31)	0.000	–0.24 (–0.40, –0.09)	0.002	–0.22 (–0.37, –0.06)	0.006	–0.29 (–0.44, –0.14)	0.000	–0.11 (–0.26, 0.04)	0.155		
Tuberculosis vaccination (ref: vaccinated)												
*Not vaccinated*	–0.41 (–0.56, –0.27)	0.000	–0.10 (–0.26, 0.05)	0.193			–0.21 (–0.37, –0.05)	0.010	0.03 (–0.13, 0.20)	0.675		
Malnutrition screening (ref: child screened)												
*Child not screened*	–0.01 (–0.15, 0.14)	0.930	0.00 (–0.13, 0.14)	0.961			–0.16 (–0.31, 0.00)	0.051	–0.09 (–0.24, 0.06)	0.262		
Health-related knowledge (ref: high)												
*Low-to-moderate*	–0.12 (–0.27, 0.04)	0.142	0.03 (–0.12, 0.17)	0.739			–0.04 (–0.20, 0.12)	0.628	0.09 (–0.07, 0.25)	0.264		
**Immediate level**												
Minimum dietary diversity (ref: meets)												
*Does not meet*	–0.43 (–0.58, –0.28)	0.000	–0.10 (–0.26, 0.06)	0.231			–0.27 (–0.43, –0.12)	0.001	0.02 (–0.16, 0.20)	0.818		
Fruits/vegetable intake the previous day (ref: consumed)												
*Did not consume*	–0.43 (–0.58, –0.29)	0.000	–0.03 (–0.19, 0.14)	0.758			–0.25 (–0.40, –0.10)	0.001	–0.00 (–0.18, 0.18)	0.999		
Vitamin A food intake the previous day (ref: consumed)												
*Did not consume*	–0.31 (–0.46, –0.16)	0.000	–0.00 (–0.16, 0.15)	0.977			–0.24 (–0.39, –0.09)	0.002	–0.07 (–0.23, 0.09)	0.404		
Iron-rich food intake the previous day (ref: consumed)												
*Did not consume*	–0.25 (–0.40, –0.10)	0.001	–0.07 (–0.22, 0.07)	0.323			–0.16 (–0.32, –0.01)	0.040	–0.01 (–0.17, 0.15)	0.906		
Egg/flesh food intake the previous day (ref: consumed)												
*Did not consume*	–0.23 (–0.37, –0.08)	0.002	–0.05 (–0.20, 0.09)	0.472			–0.40 (–0.56, –0.25)	0.000	–0.23 (–0.40, –0.06)	0.009	–0.20 (–0.38, –0.02)	0.028
Sugary food intake the previous day (ref: not consumed)												
*Consumed*	0.13 (–0.02, 0.28)	0.092	0.01 (–0.13, 0.15)	0.897			0.12 (–0.03, 0.28)	0.110	0.03 (–0.12, 0.18)	0.720		
Illness in the last 2 weeks (ref: no illness)												
*Illness*	–0.10 (–0.25, 0.06)	0.228	–0.15 (–0.29, –0.00)	0.049			–0.13 (–0.30, 0.03)	0.120	–0.13 (–0.28, 0.03)	0.114		
Maternal MUAC (ref: 24–29.5 cm)												
*Underweight (<24 cm)*	–0.24 (–0.45, –0.02)	0.030	–0.23 (–0.43, –0.03)	0.022	–0.21 (–0.41, –0.02)	0.032	–0.00 (–0.20, 0.20)	0.997	0.00 (–0.18, 0.19)	0.959	0.02 (–0.16, 0.20)	0.804
*Overweight (>29.5 cm)*	0.18 (0.01, 0.35)	0.040	0.15 (–0.01, 0.30)	0.071	0.12 (–0.04, 0.28)	0.139	0.20 (0.02, 0.38)	0.030	0.22 (0.05, 0.39)	0.012	0.21 (0.04, 0.39)	0.016
Maternal age (ref: 24–34 y)												
*Younger (<24)*	–0.01 (–0.23, 0.21)	0.932	–0.09 (–0.29, 0.11)	0.376			0.08 (–0.15, 0.31)	0.497	–0.01 (–0.23, 0.21)	0.922		
*Older (≥35)*	–0.01 (–0.17, 0.15)	0.896	0.04 (–0.11, 0.19)	0.577			–0.01 (−0.18, 0.15)	0.893	0.03 (–0.13, 0.19)	0.733		

CI – confidence interval, CU5 – children under five, HHS – household hunger scale, FCS – food consumption score, M2M – mother-to-mother, MUAC – mid-upper arm circumference, NA – not assessed, NFI – non-food item, rCSI – reduced coping strategy index, ref – reference

*The final adjusted models may have a smaller sample size due to a few variables missing some observations.

†Adjusting for child age, sex, household region, and trial arm.

‡Corresponding to Integrated Food Security Phase Classification 1/2 (*i.e.* minimal/stressed).

§Corresponding to Integrated Food Security Phase Classification 3/4/5 (*i.e.* crisis/emergency/famine).

At the intervention level, not attending M2M support groups was positively associated with WHZ in the endline overall model (*β* = 0.17; 95% CI = 0.01, 0.33), likely due to reverse causality (**Table 3**).

At the underlying level, having more than one CU5 in the household was protective of WHZ (*β* = 0.24; 95% CI = 0.07, 0.40), while maternal defecation in an open area *vs.* a latrine adversely affected WHZ in the overall midline final adjusted model (*β* = −0.22; 95% CI = −0.37, −0.06), and these correlates had similar effect sizes. Similarly, household disposal of children’s stool in an open area *vs.* a latrine was associated with a higher risk of wasting in the Poisson regression endline model (Table S5 in the **Online Supplementary Document**). Regarding measures of food insecurity, having a reduced coping strategy index (rCSI) score ≥19 was negatively associated with WHZ (*β* = −0.17; 95% CI = −0.33, −0.01) in the endline overall final adjusted model, although this correlate had a slightly smaller effect size magnitude than the underlying level correlates in the midline overall final adjusted model (**Table 3**).

At the immediate level, the mother being underweight adversely affected WHZ in the midline overall model (*β* = −0.21; 95% CI = −0.41, −0.02), and, similarly, the mother being overweight was protective of WHZ in the overall endline model (*β* = 0.21; 95% CI = 0.04, 0.39), with these correlates having equal effect sizes in both models at a magnitude of 0.21 SD. Not consuming eggs and/or flesh foods the previous day was associated with a lower WHZ in the endline overall adjusted model (*β* = −0.20, 95% CI = −0.38, −0.02), demonstrating a protective effect of protein-rich food consumption (**Table 3**).

When stratifying by child age, no new correlates of WHZ were identified. Instead, household region remained a statistically significant correlate in the endline 9–23-month final adjusted model and in both the midline and endline 24–59-month final adjusted models, with a large effect size ranging from 0.31 to 0.80 SD in these models. Maternal education was identified as a statistically significant correlate in the endline 9–23-month final adjusted model and having more than one CU5 was again protective of WHZ in the midline 9–23-month final adjusted model. Maternal defecation practices and egg and/or flesh foods consumption were associated with WHZ in the midline and endline 24–59-month final adjusted models, respectively, and the mother being overweight was again protective of WHZ in the endline 9–23-month final adjusted model (Tables S6–9 in the **Online Supplementary Document**).

When stratifying by region, some correlates consistent with the overall final adjusted models were identified, while a few new statistically significant correlates emerged. At midline in Hiran, having more than one CU5 in the household remained as a protective correlate of WHZ and having an underweight mother was again correlated with lower WHZ in the final adjusted model. Similarly, in Bay at endline, the mother being overweight again appeared protective of WHZ. On the other hand, maternal decision-making (where the mother was the decision-maker for income, health care, and purchases), as opposed to joint decision-making, correlated with lower WHZ at midline in Hiran and at endline in Bay, with effect sizes of 0.27 SD and 0.41 SD, respectively. In Hiran, household open defecation (*i.e.* not using a toilet facility and defecating openly in a bush or field) was negatively correlated with WHZ at midline, with a relatively large effect size of 0.40 SD, while not receiving an NFI kit (kits were distributed to the most vulnerable households) was protective at endline in the final adjusted model. In Bay, recent child illness adversely affected WHZ at midline, and living in a father- or other-headed household was associated with lower WHZ compared with maternal-headed households at endline (Tables S10–13 in the **Online Supplementary Document**).

### Correlates of mothers’ wasting

While additional correlates may have been statistically significant in the unadjusted or basic confounder-adjusted models, the final adjusted models with the hierarchical approach are presented here (Figure S6 and Tables S14 and S15 in the **Online Supplementary Document**).

Maternal household decision-making was associated with wasting at midline, with mothers living in maternal-decision-making households having a 0.49 times lower relative risk of wasting than those in joint decision-making households. Regarding intervention components, not attending M2M groups was statistically significantly associated with wasting at endline in the final adjusted model. At the underlying level at midline, having an unacceptable food consumption score and being in a household with moderate-to-severe hunger were both associated with increased relative risks of wasting, at 1.60- and 1.79-times higher, respectively. Disposing of children’s stool in an open area was associated with a 2.04 times higher relative risk of wasting at midline, and household practice of open defecation was associated with a higher risk of wasting at endline, although with a relatively smaller effect size of 1.57 times the relative risk. At the immediate level, age was statistically significantly associated with wasting at midline, with older mothers (≥35 years) having a 0.40 times lower relative risk of wasting in comparison to mothers aged 24–34 years (Tables S14 and S15 in the **Online Supplementary Document**).

## DISCUSSION

To the authors’ knowledge, this is the first study to explore the correlates of wasting among both CU5 and their mothers in two high-wasting-burden regions of Somalia. Hiran had approximately a 3–4 times higher burden of child wasting compared to Bay, and region appeared as a statistically significant correlate of child WHZ in five out of six relevant child models (excluding the region-stratified models), with relatively large effect sizes ranging from 0.31–0.80 SD, justifying stratifying the analyses by region to better understand the region-specific factors contributing to wasting. While maternal MUAC and household decision-making were statistically significant correlates in both regions, additional unique factors were identified in each region. When analyses were stratified by child age, the only common correlate across children aged 9–23 months and 24–59 months was region. However, additional factors influencing WHZ were identified for each age group, suggesting that the most significant correlates of child nutrition outcomes may differ across age groups. For example, consumption of egg and/or flesh foods was retained as a statistically significant correlate of WHZ among children aged 24–59 months at endline, but not among children under two at endline. Additionally, stratifying by child age allowed for further studying relationships initially observed in the non-stratified models; for example, while having no maternal education had a relatively small effect size of 0.17 SD in the endline overall final adjusted model, in the endline 9–23-month model, a much larger effect size of 0.59 SD was observed, demonstrating the importance of maternal education for child nutrition outcomes in this subgroup. Regarding seasonality, multiple correlates of WHZ differed between midline (Xagaa dry season/Deyr rainy season transition) and endline (Jilaal dry season), and previous work has shown that seasonality is a significant predictor of malnutrition among CU5 in Somalia [10,12,13]. Further supporting this finding, all of the statistically significant correlates of mothers’ wasting in the final adjusted models differed between midline and endline. Taken together, these findings highlight the importance of recognising the context-specific nature of correlates of child and maternal wasting in program efforts, understanding that the most significant correlates in a given context may vary by sub-population, region, and season. However, these differences in identified correlates should be interpreted with caution. Across models, we excluded indicators from specific analyses when they were highly imbalanced in the sample. As a result, regional, seasonal, and child-age differences in correlates may not fully account for all relevant factors.

Multiple characteristics of maternal empowerment were associated with child nutrition status. The mother having no formal education was associated with lower WHZ in different stratified CU5 models, consistent with previous work in Ethiopia, where maternal illiteracy and lack of education were risk factors for wasting among children aged 6–59 months [43–45]. Regarding household decision-making, different results were found between children and mothers. Not having joint household decision-making between both parents was associated with lower child WHZ, and these findings are consistent with work done in Ethiopia, where children in households with maternal-only or paternal-only decision-making were at an increased risk of wasting compared to children in joint decision-making households [45]. On the other hand, maternal decision-making was found to be a protective factor against maternal wasting compared to joint household decision-making, aligning with findings from studies in Ethiopia where household decision-making and control were predictive of maternal nutrition status [46–48]. While further work in this context is needed to explore these findings, as one hypothesis for these results, husbands’ involvement in joint decision-making may reflect more involvement in childcare and therefore be beneficial for child nutrition; for example, a study in Nepal found that joint decision-making reflected greater husband involvement in pregnancy care [49,50]. A possible explanation for the protective effect of maternal-only decision-making on maternal wasting is that mothers can prioritise their own health more effectively, aligning with studies that link greater autonomy to better health service utilisation [47,51].

Not receiving an NFI kit was protective of WHZ. As the most vulnerable households were selected to receive NFI kits, this result supports the idea that those who did not receive an NFI kit were less vulnerable and likely better protected against wasting. Additionally, the mother not attending M2M groups showed a protective relationship with both child WHZ (although at a smaller effect size relative to other reported correlates) and maternal wasting at endline. However, this finding does not suggest that M2M groups were harmful, but rather, as this variable is an intervention component measured in a cross-sectional survey, the phenomenon of reverse causality is likely, where those who receive intervention components (in this case, attending M2M groups) may be the most vulnerable and already have poorer nutrition outcomes [52–57]. Mothers who did not attend M2M groups were either those who reported that M2M groups were available in the area but did not attend, or those who reported that M2M groups were not available in their area. The understanding of this finding is not that attending the groups contributes to poor nutrition outcomes. Instead, the hypothesis is that individuals with poor outcomes were perhaps more likely to attend the groups to benefit from the messaging, support, and socialisation with other mothers (*i.e.* perhaps vulnerable mothers were more incentivised by the belief that they would benefit from the groups) and/or that the communities with M2M groups available were more vulnerable and had poorer outcomes than some of the communities where M2M groups were not formed.

Multiple sanitation indicators were found to correlate with wasting, consistent with previously identified factors [32,58]. Poor sanitation practices, such as open defecation and improper disposal of stool, were identified as risk factors for both maternal and child wasting. These practices likely increase the risk of infection and illness, which are key factors contributing to wasting [10-13,58–60]. These results emphasise the importance of improving sanitation practices in this context, such as by improving infrastructure to increase household access to latrines.

Food security is an established risk factor for wasting, and household food security has previously been identified as a significant determinant of nutritional status among pregnant women in Ethiopia in multiple studies [32,46,59,61–63]. We examined three measures of household food security: the household hunger scale, the food consumption score, and the rCSI [64–66]. Consistent with previous literature identifying household food security as a determinant of women’s nutrition status, both an unacceptable food consumption score and moderate-to-severe household hunger were risk factors for mothers’ wasting in this analysis [46,61–63,67]. Similarly, at endline, a higher rCSI score, corresponding to crisis/extreme/famine levels of food insecurity, was correlated with lower child WHZ, although with a smaller effect size than some of the other correlates reported here [68]. Nevertheless, since wasting is defined based on a specific WHZ threshold, even a small change in child WHZ can determine whether a child is classified as wasted and referred to treatment, and, therefore, these findings emphasise promoting household food security to support the nutrition status of children and mothers in this setting, such as by ensuring access to local markets equipped with affordable and nutritious foods [31].

Having more than one CU5 in a household was protective of WHZ across multiple models, contrary to previous analyses of Somalia household survey data, which found that having more CU5 in a household was associated with a higher risk of child wasting [10,12,13]. However, data from Uganda showed that having siblings was protective of infant stunting, demonstrating that having multiple children may be protective of child nutrition status [69]. As this correlate emerged as statistically significant in multiple final adjusted models with an effect size ranging from 0.24–0.34 SD, this relationship between the number of CU5 in a household and child WHZ warrants additional research in this context.

In addition to household region, maternal MUAC was another frequently observed statistically significant correlate of WHZ across models, with effect sizes ranging from 0.21–0.48 SD in the final adjusted models. Maternal malnutrition is a cited risk factor for child wasting, emphasising the close relationship between maternal characteristics and child nutrition outcomes [59]. Our results are consistent with previous work in Somalia, where higher maternal MUAC was associated with lower wasting among children aged 6–59 months [11–13]. The association between maternal MUAC and child WHZ demonstrates the intergenerational and interconnected nature of maternal and child nutrition status and supports the design of policies and programs to protect mothers' nutrition status [59,70,71].

Consumption of egg and/or flesh foods was protective of WHZ. Animal-based foods reflect improved nutrient intake for optimal child growth, and these findings are consistent with previous work in Somalia, where protein- and animal-source foods were important for the nutritional status of CU5 [10–13,72]. These findings highlight the importance of ensuring that high-protein, animal-based foods are available in local markets and that households have the financial means to afford such foods.

### Strengths and limitations

Limitations of our study include the potential for recall bias and the potential effect of unmeasured confounders. Additional limitations include data for some variables only being available at one time point, not being able to include some indicators in specific models due to high imbalance in the analysis sample, and the questionnaire not including questions about maternal diet and disease, limiting the ability to analyse important immediate determinants of maternal nutrition status. While outside our scope, which sought to remain true to the original data, future analyses could explore imputation methods, such as multiple imputation, to address the limitation that some indicators were not included in specific models due to a high imbalance in our sample. Nevertheless, the strengths of our study include adequate sample sizes and the ability to analyse a wide breadth of indicators spanning the entire conceptual framework of maternal and child nutrition, to study the relationship between maternal and child nutrition, and to implement a backwards elimination multivariable model-building approach in a hierarchical way to arrive at fully adjusted models that minimise the effects of confounding. Additionally, although our study was a cross-sectional analysis, the data were from a prospective cohort study, which allowed comparisons of the correlates of wasting at two different time points corresponding to different seasons, and we used stratified models to study associations within strata of key confounding variables [73]. While causality cannot be established from a cross-sectional analysis, we selected covariates based on biological plausibility and consistency with existing knowledge, and the statistically significant findings from the fully adjusted models demonstrate the strength of association of the identified correlates [73–75]. Although cross-sectional analysis does not allow for examining direct temporal relationships, wasting is a dynamic condition that can change quickly [32,73–75]. By analysing factors present before assessing participants' nutritional status – such as illness in the past 2 weeks or vaccination status – some insights into exposure history can still be inferred [75]. While we used a hierarchical approach to consider basic/enabling, underlying, and immediate-level factors affecting nutrition, future analyses could use statistical methods such as LASSO or AIC-based stepwise methods to assess the comparability of findings.

### Future research

Findings are generalisable to similar such humanitarian contexts (such as conflict- and climate-affected contexts) in Somalia and other countries, and additional research is needed to further explore the identified differences in correlates between groups and across seasons. Future research should examine the correlates of maternal and child wasting in other high-wasting-burden areas of Somalia, as well as across different seasons, to construct a more complete picture of how the most significant correlates of wasting may fluctuate throughout the year. Future research should also examine characteristics of maternal diet and disease as potential correlates of mothers’ wasting, further explore the association between the number of CU5 in a household and child wasting outcomes, and further study gender dynamics in this context and implications across regions and seasons for nutrition outcomes, household resources, food and nutrition practices, and health-seeking behaviours. Given the differing effects of household decision-making dynamics on child and mother nutrition outcomes in this study, additional survey questions to better understand these dynamics should be included in future studies, and qualitative research should further explore them in this context.

## CONCLUSIONS

To address the high burden of wasting in Somalia, it is important to explore the most significant correlates of maternal and child wasting. We highlight regional, age, and seasonal variation in the correlates of wasting, underscoring the importance of context-specific approaches to addressing malnutrition. Key correlates, such as maternal MUAC and household decision-making, were consistent across regions, while others, including child illness, food security, and animal-based protein consumption, varied by region, season, and age group. The findings emphasise the need for tailored interventions that account for local factors, including household food security and sanitation practices, seasonality, and gender dynamics. We also highlight the interconnectedness of maternal and child nutritional status, reinforcing the importance of maternal health in improving child nutrition outcomes.

Future research should further explore the nature and implications of gender dynamics in this setting for maternal and child nutrition, and characterise the correlates of wasting during other seasons and in other high-wasting-burden areas of Somalia. These findings contribute to a growing body of literature on the complex, multifactorial correlates of wasting and underscore the importance of designing multisectoral interventions that address the immediate, underlying, and basic causes of malnutrition.

## Additional material


Online Supplementary Document
