# Supplementary material for: Assessing the correlates of wasting among children under five and their mothers in the Bay and Hiran regions of Somalia
Source: J Glob Health. 2025 Dec 12;15:04308. doi: 10.7189/jogh.15.04308 (PMC12699499; doi:10.7189/jogh.15.04308)
Supplement: Online Supplementary Document [file jogh-15-04308-s001.pdf]

Supplement to: Grounds S, Walton S, Alier KK, Garretson S, Mohamoud SA, Abdiqadir SM, Khattak Q, Nur MA, Mohamoud AM, Ismail MO, Mahat MB, Mohamed AA, Mohamed AA, Tripaldi M, Akseer N. Assessing the Correlates of Wasting among Children Under 5 and Their Mothers in The Bay and Hiran Regions of Somalia. J Glob Health. 2025;15:04308.

Authors: Samantha Grounds[1], Shelley Walton[1], Kemish Kenneth Alier[1], Sydney Garretson[1], Said Aden Mohamoud[2], Sadiq Mohamed Abdiqadir[2], Qundeel Khattak[3], Mohamud Ali Nur[2], Abdullahi Muse Mohamoud[4], Meftuh Omer Ismail[2], Mohamed Billow Mahat[2], Adam Abdulkadir Mohamed[2], Abdifatah Ahmed Mohamed[4], Marina Tripaldi[3], Nadia Akseer[1]

[1]Johns Hopkins University, Department of International Health, Baltimore, United States of America

[2]Save the Children, Somalia Country Office, Mogadishu, Somalia

[3]Save the Children, Save the Children International, London, United Kingdom

[4]Federal Government of Somalia, Ministry of Health & Human Service, Mogadishu, Somalia

Supplementary Materials

Table of Contents

Appendix S1. Technical Appendix

- **Text S1:** Technical Definition of Wasting
- **Text S2:** Somalia CashPlus for Nutrition cRCT Overview
- **Text S3:** Study Setting and Context
- **Text S4:** The Strengthening the Reporting of Observational Studies in Epidemiology (STROBE) Checklist
- **Table S1.** Independent Variables Tested as Correlates of CU5 and Mothers' Wasting
- **Table S2:** Definitions of Key Indicators
- **Text S5:** Construction of Different Indicators
- **Figure S1.** Mapping of Variables Tested in the Mothers' Models
- **Text S6:** Defining the Study Sample
- **Text S7:** Modeling Strategy

Appendix S2. Results Appendix

- **Figure S2.** Flow Diagram for the Midline CU5 Sample
- **Figure S3.** Flow Diagram for the Endline CU5 Sample
- **Figure S4.** Flow Diagram for the Midline Mother Sample
- **Figure S5.** Flow Diagram for the Endline Mother Sample
- **Table S3.** Additional Child, Mother, and Household Characteristics
- **Table S4.** Midline Overall CU5 Model: Correlates of Wasting
- **Table S5.** Endline Overall CU5 Model: Correlates of Wasting
- **Table S6.** Midline 9-23 Months Model: Correlates of Weight-for-Height Z-Score
- **Table S7.** Midline 24-59 Months Model: Correlates of Weight-for-Height Z-Score
- **Table S8.** Endline 9-23 Month CU5 Model: Correlates of Weight-for-Height Z-Score

Table S9. Endline 24-59 Month CU5 Model: Correlates of Weight-for-Height Z-Score
Table S10. Midline Hiran Region CU5 Model: Correlates of Weight-for-Height Z-Score
Table S11. Midline Bay Region CU5 Model: Correlates of Weight-for-Height Z-Score
Table S12. Endline Hiran Region CU5 Model: Correlates of Weight-for-Height Z-Score
Table S13. Endline Bay CU5 Model: Correlates of Weight-for-Height Z-Score
Figure S6. Correlates of Mothers' Wasting
Table S14. Midline Overall Maternal MUAC Model: Correlates of Wasting
Table S15. Endline Overall Maternal MUAC Model: Correlates of Wasting

Appendix S1. Technical Appendix

Text S1: Technical Definition of Wasting

Wasting among children is defined by a weight-for-height Z-score (WHZ) measurement < -2 standard deviations (SD) from the 2006 WHO Child Growth Standards median and is further characterized as either moderate (< -2 but ≥ -3 SD) or severe (< -3 SD).[1–6] Wasting among mothers is defined as a mid-upper arm circumference (MUAC) of less than 23 centimeters.[7–9]

Text S2: Somalia CashPlus for Nutrition cRCT Overview

Data for this study comes from a prospective, mixed methods cluster randomized controlled trial (cRCT) to evaluate the effectiveness of a CashPlus for Nutrition program implemented by Save the Children (SC) in the Bay and Hiran regions of Somalia from June-November 2023.[10,11] The specific study sites were the capitals of these regions, Baidoa (Bay) and Beledweyne (Hiran). The CashPlus for Nutrition project was aimed at preventing acute malnutrition among CU5 and their mothers through various combinations of cash and social and behavior change communication (SBCC) interventions.[10,11]

The cRCT lasted for six months, and villages were randomized to three study arms: 1) cash only, 2) cash plus SBCC, and 3) cash plus additional cash top-up.[10,11] Participants were mothers of children under 5 (CU5) enrolled in the CashPlus for Nutrition program who were contacted and recruited for participation in the trial.[10,11] Participants received cash monthly via a mobile transfer, with participants in Bay receiving an amount equivalent to 90 US dollars (USD) and participants in Hiran receiving an amount equivalent to 70 USD.[10,11] Those randomized to the cash plus additional cash top-up trial arm received an additional 35 USD per month.[10,11] Participants randomized to the cash plus SBCC trial arm received community-level promotion campaigns, individual sessions, and mother-to-mother (M2M) group sessions around health and nutrition-related issues.[10,11] Additional details of the cRCT are published elsewhere.[10,11] For the purposes of this study, the cRCT trial arm was adjusted for in the analysis to control for any effect of the interventions.

Text S3: Study Setting & Context

Somalia is found in the east of the Horn of Africa and is characterized by a tropical, mediterranean climate.[12,13] Somalia's calendar year is characterized by four seasons: two rainy seasons and two dry seasons.[12] Jilaal dry season occurs from December to March, followed by the Gu rainy season that occurs in the latter half of March through June.[14] The Gu rainy season accounts for the majority of the country's rainfall, at about 75% of total rainfall.[14] The Xagaa dry season then takes place from July through September, with some rainfall occurring in certain parts of the country.[12,14] Finally, the Deyr rainy season occurs from September through November, accounting for about a quarter of the country's annual rainfall.[12,14] Over the years, climate change has disrupted typical weather patterns, resulting in repeated droughts and flooding throughout Somalia.[12]

Climate-related issues (droughts, flooding) and conflict have contributed to issues of food insecurity and displacement, causing Somalia to be characterized as a "complex emergency."[15]

It is estimated that nearly six million individuals in Somalia will require humanitarian assistance in 2025, and Somalia is estimated to have 3.3 million internally displaced people (IDPs).[15] Approximately 4.4 million individuals were also expected to experience acute food insecurity from October-December 2024.[15]

This study took place in the Bay and Hiran regions of Somalia, found in the southwest region of the country.[16–19] Recent data estimates a population of approximately 1.3 million people in Bay and roughly 0.5 million people in Hiran, with the majority of the population in both regions being rural, but with Hiran having a higher percentage of individuals living in urban areas compared to Bay.[20] Consistent with the situation seen throughout the country, flooding, droughts, and conflict have threatened the livelihoods of individuals in Bay and Hiran.[17–19,21–23] As of December 2023, more than 100,000 people in both Bay and Hiran regions (separately) were reported to have been affected by the flooding that began in Somalia in October 2023.[21]

Frequent droughts and flooding have contributed to both displacement from these regions as well as arrivals of IDPs to these regions throughout the years.[17–19,22,23] For example, while over a quarter of a million individuals were displaced from Hiran from July 2021-November 2022, close to half a million newly displaced people arrived in Hiran in 2023.[19, 24] Similar trends are seen in Bay, where thousands of individuals have been both displaced from and arrived in the Bay region as a result of such instability.[17,19,22–24] Baidoa city in Bay is home to approximately 25% of Somalia's IDP sites, with 0.75 million Bay residents experiencing crisis or emergency levels of food insecurity from January-March 2023.[19,23]

Text S4: The Strengthening the Reporting of Observational Studies in Epidemiology (STROBE) Checklist [25]

	**Item No**	**Recommendation**	**Page No**
**Title and abstract**	1	(*a*) Indicate the study's design with a commonly used term in the title or the abstract	1
		(*b*) Provide in the abstract an informative and balanced summary of what was done and what was found	1
**Introduction**			
Background/rationale	2	Explain the scientific background and rationale for the investigation being reported	1-2
Objectives	3	State specific objectives, including any prespecified hypotheses	2
**Methods**			
Study design	4	Present key elements of study design early in the paper	2-4

Setting	5	Describe the setting, locations, and relevant dates, including periods of recruitment, exposure, follow-up, and data collection	2-3; Text S3
Participants	6	(*a*) Give the eligibility criteria, and the sources and methods of selection of participants	4; Text S6
Variables	7	Clearly define all outcomes, exposures, predictors, potential confounders, and effect modifiers. Give diagnostic criteria, if applicable	3; Text S1; Table S1-S2
Data sources/ measurement	8*	For each variable of interest, give sources of data and details of methods of assessment (measurement). Describe comparability of assessment methods if there is more than one group	3; Table S2; Text S5
Bias	9	Describe any efforts to address potential sources of bias	3-4; Text S7
Study size	10	Explain how the study size was arrived at	3-4; Text S6
Quantitative variables	11	Explain how quantitative variables were handled in the analyses. If applicable, describe which groupings were chosen and why	3-4; Table S1-2; Text S5; Text S7
Statistical methods	12	(*a*) Describe all statistical methods, including those used to control for confounding	3-4; Text S7
		(*b*) Describe any methods used to examine subgroups and interactions	3-4; Text S7
		(*c*) Explain how missing data were addressed	3-4; Text S6-7
		(*d*) If applicable, describe analytical methods taking account of sampling strategy	N/A
		(*e*) Describe any sensitivity analyses	N/A
**Results**			
Participants	13*	(a) Report numbers of individuals at each stage of study—eg numbers potentially eligible, examined for eligibility, confirmed eligible,	5; Figure S2-S5

		included in the study, completing follow-up, and analysed	
		(b) Give reasons for non-participation at each stage	N/A
		(c) Consider use of a flow diagram	Figure S2-S5
Descriptive data	14*	(a) Give characteristics of study participants (eg demographic, clinical, social) and information on exposures and potential confounders	5; Table 1; Table S3
		(b) Indicate number of participants with missing data for each variable of interest	5; Table 1; Table S3
Outcome data	15*	Report numbers of outcome events or summary measures	5; Table 2
Main results	16	(*a*) Give unadjusted estimates and, if applicable, confounder-adjusted estimates and their precision (eg, 95% confidence interval). Make clear which confounders were adjusted for and why they were included	5-11; Table 3; Table S4-15
		(*b*) Report category boundaries when continuous variables were categorized	Table 3; Table S4-15
		(*c*) If relevant, consider translating estimates of relative risk into absolute risk for a meaningful time period	N/A
Other analyses	17	Report other analyses done—eg analyses of subgroups and interactions, and sensitivity analyses	10; Table S6-13
**Discussion**			
Key results	18	Summarise key results with reference to study objectives	11-13
Limitations	19	Discuss limitations of the study, taking into account sources of potential bias or imprecision. Discuss both direction and magnitude of any potential bias	13
Interpretation	20	Give a cautious overall interpretation of results considering objectives, limitations, multiplicity	14

		of analyses, results from similar studies, and other relevant evidence	
Generalisability	21	Discuss the generalisability (external validity) of the study results	13
**Other information**			
Funding	22	Give the source of funding and the role of the funders for the present study and, if applicable, for the original study on which the present article is based	14

Table S1. Independent Variables Tested as Correlates of CU5 and Mothers' Wasting

Area of Interest	Independent Variable	Categories (Coding)	Reference Category
Location	Region	Bay (0), Hiran (1)	Bay
Wealth and Income Poverty	Wealth/Asset Index	Not Poor (0), Poor (1)	Not Poor
	Health- and Nutrition-Related Monthly Expenditure	Middle 50% (0), Lower 25% (1), Upper 25% (2)	Middle 50%
Displacement	Displacement in the Last 3 Months	Not Displaced (0), Displaced (1)	Not Displaced
Household Decision-Making and Women's Empowerment	Head of Household	Mother (0), Father/Other (1)	Mother
	Household Decision-Making	Joint (0), Maternal (1), Paternal (2)	Joint
	Maternal Education*	Some Education (0), No Education (1)	Some Education
Intervention Components	Mother-to-Mother Groups	Attended (0), Did not Attend (1)	Attended
	Non-Food Item Kits†	Received (0), Did not Receive (1)	Received
Household Food Security	Household Hunger Scale	Little-to-No Hunger (0), Moderate-to-Severe Hunger (1)	Little-to-No Hunger
	Food Consumption Score	Acceptable (>35) (0), Unacceptable (≤35) (1)	Acceptable
	Reduced Coping Strategy Index	Score of <19 (0), Score of ≥19 (1)	Score of <19
	Number of CU5	1 Child (0), 2+ Children (1)	1 Child
	Household Crowding	Not Crowded (0), Crowded (1)	Not Crowded

Household Environment	Water Treatment	Treats (0), Does not Treat (1)	Treats
	Water Source	Piped (0), Not Piped (1)	Piped
	Household Defecation	Latrine/Toilet (0), Open Defecation (1)	Latrine/Toilet
	Child Stool Disposal	Latrine/Toilet (0), Open Area (1)	Latrine/Toilet
	Maternal Defecation Practices	Latrine/Toilet (0), Open Area (1)	Latrine/Toilet
Health- and Nutrition-Related Knowledge and Practices	Tuberculosis Vaccination‡	Vaccinated (0), Not Vaccinated (1)	Vaccinated
	Child Malnutrition Screening‡	Child Screened (0), Child Not Screened (1)	Child Screened
	Maternal Malnutrition Screening§	Mother Screened (0), Mother Not Screened (1)	Mother Screened
	Health-Related Knowledge	High (0), Low-to-Moderate (1)	High
Child Diet‡	Ever Breastfed	Breastfed (0), Not Breastfed (1)	Breastfed
	Minimum Dietary Diversity	Meets (0), Does not Meet (1)	Meets
	Fruit/Vegetable Consumption	Consumed (0), Did not Consume (1)	Consumed
	Vitamin A-Rich Food Consumption	Consumed (0), Did not Consume (1)	Consumed
	Iron-Rich Food Consumption	Consumed (0), Did not Consume (1)	Consumed
	Egg and/or Flesh-Food Consumption	Consumed (0), Did not Consume (1)	Consumed
	Sugary Food Consumption	Did not Consume (0), Consumed (1)	Did not Consume
Child Disease‡	Illness in the Last 2 Weeks	Not Ill (0), Ill (1)	Not Ill
Maternal Characteristics	Maternal MUAC‡	Normal (24-29.5 cm) (0), Underweight (<24 cm) (1), Overweight (≥ 29.5 cm) (2)	Normal
	Maternal Age	Middle (24-34) (0), Younger (<24) (1), Older (35+) (2)	Middle

*For maternal education, "some education" included madrasa, primary school, secondary school, and higher education.

†The most vulnerable households were selected to receive WASH non-food item/hygiene kits according to a set of criteria determined by Save the Children Somalia country office.
‡These variables were only tested in the CU5 models.
§This variable was only tested in the mothers' models.

Table S2: Definitions of Key Indicators

Child-Related Key Indicators		
**Indicator**	**Numerator**	**Denominator**
Child Illness in the Past 2 Weeks	Children in the study sample having diarrhea, fever, and/or a cough in the 2 weeks preceding the survey	Children in the study sample
Tuberculosis Vaccination	Children in the study sample ever having received the tuberculosis vaccination (BCG against TB)	Children in the study sample
Child Malnutrition Screening	Children in the study sample having their height, weight, or arm circumference measured in a facility, in the community, or at home in the last 3 months	Children in the study sample
Child Ever Breastfed	Children in the study sample where the mother reported that the child has ever been breastfed[26]	Children in the study sample
Minimum Dietary Diversity 7 (MDDC7)	Children in the study sample consuming at least 4/7 of the following food groups (as a food or beverage) the previous day or night preceding the survey: grains, roots, tubers; pulses, nuts, seeds; dairy products; flesh foods; eggs; vitamin A-rich fruits and vegetables; other fruits and vegetables[27–29]	Children in the study sample
Minimum Dietary Diversity 8 (MDDC8)	Children aged 9-23 months in the study sample consuming at least 5/8 of the following food groups (as a food or beverage) the previous day or night preceding the survey: breastmilk; grains, roots, tubers; pulses, nuts, seeds; dairy products; flesh foods; eggs; vitamin A-rich fruits and vegetables; other fruits and vegetables[27–29]	Children aged 9-23 months in the study sample
Egg and/or Flesh Food Consumption	Children in the study sample consuming any eggs and/or animal-based flesh foods the previous day or night preceding the survey[29,30]	Children in the study sample
Fruit/Vegetable Consumption	Children in the study sample consuming any fruit and/or vegetables	Children in the study sample

	in the previous day or night preceding the survey[29,31]	
Vitamin A-Rich Food Consumption	Children in the study sample consuming any of the following in the previous day or night preceding the survey: any vitamin A-rich fruits, tubers, or vegetables, dark green leafy vegetables, red palm oil foods, eggs, milk products, and/or animal organ meat[32]	Children in the study sample
Iron-Rich Food Consumption	Children in the study sample consuming any of the following in the previous day or night preceding the survey: dark green leafy vegetables, legumes, nuts, fish/seafood and/or any animal flesh foods[33]	Children in the study sample
Sugary Food Consumption	Children in the study sample consuming any sugary foods (chocolates/candies, cakes, pastries, etc.) the previous day or night preceding the survey[29,34]	Children in the study sample
	Intervention Key Indicators	
Indicator	***Numerator***	***Denominator***
Attending M2M Groups	Households in the study sample where the mother reported attending a mother-to-mother support group session	Households in the study sample
Receiving NFI Kits	Households in the study sample where the mother reported that the household received a non-food item kit	Households in the study sample
	Maternal- and Household-Related Key Indicators	
**Indicator**	**Numerator**	**Denominator**
Household Decision-Making	Maternal: Households in the study sample where the mother was the decision-maker for income, healthcare, and purchases<br>Paternal: Households in the study sample where the father was the decision-maker for income, healthcare, and purchases<br>Joint: Households in the study sample where there was any combination of mother and father decision-making for income, healthcare, and purchases	Households in the study sample

Head of Household	Mother: Households in the study sample where the mother is the reported head of household<br>Father or Other: Households in the study sample where the father or another household member (not the mother) is the reported head of household	Households in the study sample
Displacement	Households in the study sample reporting displacement in the last three months	Households in the study sample
Wealth/Asset Index[35]	Not Poor: Households in the study sample with wealth/asset indices falling into the middle, rich, or richest quintiles<br>Poor: Households in the study sample with wealth/asset indices falling in the poor or poorest quintile	Households in the study sample
Health- and Nutrition-Related Expenditure	Households in the study sample falling in the lower 25%, middle 50%, or upper 50% distribution when dividing household monthly spending on nutritious foods, medicine, and maternal and child health services by total household monthly expenditure and multiplying by 100	Households in the study sample
Maternal Education	Some Education: Households in the study sample where the mothers' current education level was reported as "Madrasa," "primary," "secondary," or "higher"<br>No Education: Households in the study sample where the mothers' current education level was reported as "none"	Households in the study sample
Household Reduced Coping Strategy Index [36]	Households in the study sample scoring a reduced coping strategy index of <19 or ≥19	Households in the study sample
Household Crowding	Households in the study sample scoring a crowding index of at least 5, according to the number of individuals living in the household divided by the number of rooms in the household[37]	Households in the study sample
Household Food Consumption Score (FCS) [38]	Acceptable: Households in the study sample with a FCS score of >35	Households in the study sample

	Unacceptable: Households in the study sample with a FCS score ≤35	
Household Hunger Scale (HHS) [39]	Little-to-No Hunger: Households in the study sample with a HHS score of 0-1<br>Moderate-to-Severe Hunger: Households in the study sample with a HHS score of 2-6	Households in the study sample
Number of Children Under 5	1 Child: Households in the study sample reporting only 1 child under 5 years of age currently living in the household<br>2+ Children: Households in the study sample reporting 2 or more children under 5 years of age currently living in the household	Households in the study sample
Household Water Source	Piped: Households in the study sample for which the main source of drinking water for individuals in the household was from piped water or a public tap<br>Other: Households in the study sample for which the main source of drinking water for individuals in the household was one of the following: borehole or tube well, protected dug well or protected spring, unprotected dug well or unprotected spring, surface water (river, lake, stream, etc.), rainwater collection, tanker truck or cart, bottled water, or other	Households in the study sample
Use of Water Treatment	Households in the study sample for which the mother reports using at least one of the following water treatment methods prior to consumption/drinking of water: boil, chlorinate, keep under the sun, filter, use aquatab[40]	Households in the study sample
Household Open Defecation	Households in the study sample having no toilet facility and relying on bush/field for defecation[41]	Households in the study sample
Household Disposal of Child's Stool	Latrine/Toilet: Households in the study sample for which the mother reports disposing of the child's stool in a latrine/toilet[42]<br>Open Area: Households in the study sample for which the mother reports disposing of the child's stool in the	Households in the study sample

	bush, an open field, the compound, or elsewhere	
Maternal Defecation Practices	Open Area: Households in the study sample for which the mother reports defecating in a bush, open field, the compound, or elsewhere<br>Latrine/Toilet: Households in the study sample for which the mother reports using a latrine/toilet for defecation	Households in the study sample
Mother Malnutrition Screening	Households in the study sample where mothers reported having their height, weight, or arm circumference measured in a facility, in the community, or at home in the last 3 months	Households in the study sample
Maternal Health-Related Knowledge	High: Households in the study sample where the mother had a score of 5-6 on the correct knowledge of the following 6 areas: exclusive breastfeeding, timing of breastfeeding initiation, timing of liquids initiation, timing of solid food initiation, handwashing moments, and water treatment methods<br>Low-to-Moderate: Households in the study sample where the mother had a score of 0-4 on the above areas of health-related knowledge	Households in the study sample

Text S5. Construction of Different Indicators

Wealth/Asset Index: A wealth/asset index was calculated, incorporating multiple indicators representative of wealth in the study area.[35] From the questionnaire, ownership of livestock (camels, cattle, goats, donkeys, horses, poultry), household assets (electricity, radio, TV, telephone, computer, refrigerator, internet, air conditioning), and personal items (watch, mobile phone, bicycle, scooter, donkey cart, truck, canoe, tractor, ox plough) as well as household floor, roofing, and wall materials were considered.[10]

Adapted binary variables were created for wealthier versus poorer floor, roofing, and wall materials. Ceramic, cement, wood, metal, and shingles roofs were considered wealthier for the purposes of this analysis, whereas having no roof or roofs constructed with leaves, grass, rustic mats, bamboo, wood planks, cardboard, cloth tent, or other materials were categorized as poorer.[12,43] Walls constructed of cement, stone, brick, cement blocks, wood planks/shingles, and covered adobe were considered wealthier in this analysis, whereas having no walls or walls constructed of cane, dirt, bamboo-mud, stone-mud, uncovered adobe, plywood, carboard, reused wood, or other materials were considered poorer.[12,43] Finally, polished, asphalt, tile, cement,

and carpet floors were considered wealthier, whereas floors of earth, dung, wood planks, bamboo, or other materials were considered poorer.[12,43]

Only characteristics that were true of <95% or >5% of the study sample were considered (for example, if only 3% of the sample owned camels, camel ownership was excluded from construction of the wealth/asset index).[43] As the study sample was different for the CU5 and mothers' models at midline and the CU5 and mothers' models at endline, the wealth/asset index was calculated separately for each of these groups (calculated 4 times). Once variables to be considered were identified according to the described threshold, principal component analysis was performed with an acceptable minimum KMO threshold of 0.5.[44] Any variables falling below this threshold were excluded in a stepwise manner, with the variable with the lowest KMO value being excluded first until all variables were above the 0.5 KMO threshold. Quintiles were constructed, categorizing households into poorest, poor, middle, rich, and richest categories.[35,45] After examining the distribution of these quintiles in each study sample, a final binary variable of poor (combining the poor and poorest quintiles) and not poor (combining the middle, rich, and richest categories) was created.

The diagram below shows what variables were retained in each sample's wealth/asset index:

**Sample**	**Variables in Final Calculated Wealth/Asset Index**
Midline CU5 Models	*Livestock Ownership:* Goats<br>*Agricultural Land Ownership*<br>*Household Assets:* Radio, Watch, Mobile Phone<br>*House Materials:* Roofing
Midline Mothers' Models	*Livestock Ownership:* Goats, Donkeys<br>*Agricultural Land Ownership*<br>*Household Assets:* Radio, Watch, Mobile Phone, Nonmobile Telephone<br>*House Materials:* Roofing, Walls
Endline CU5 Models	*Livestock Ownership:* Goats, Cattle<br>*Household Assets:* Radio<br>*House Materials:* Roofing, Walls, Flooring
Endline Mothers' Models	*Livestock Ownership:* Goats, Cattle<br>*Household Assets:* Radio<br>*Personal Assets:* Donkey Cart<br>*House Materials:* Roofing, Walls, Flooring

Health- and Nutrition-Related Expenditure: A health- and nutrition-related expenditure variable was created by summing household monthly spending on nutritious foods, medicine, and maternal and child health (MCH) services (for example, antenatal care), dividing this value by total household monthly expenditure (including spending on nutritious foods, medicine, and MCH as well as other food, hygiene, transportation, fuel, water, electricity, rent, clothes, school, agriculture, social, debt, and savings), and multiplying by 100. Summary statistics for this variable

were then examined, and a categorical variable was created to examine the lower 25%, middle 50%, and upper 25% categories.

Household Decision-Making: The questionnaire included separate questions about household decision-making on income, healthcare, and purchases.[10] In order to avoid potential collinearity if including decision-making variables for each of these three components separately, a composite household decision-making variable was created by combining the answers to each of these areas, creating joint, maternal, and paternal household decision-making categories. A household was considered to have maternal household decision-making if the mother was listed as the decision-maker in all three areas of decision-making around income, healthcare, and purchases. Similarly, a household was considered to have paternal decision-making if the father was the reported decision-maker for income, healthcare, and purchases. Any other combination of decision-making around these three separate areas was considered joint household decision-making.

Reduced Coping Strategy Index (rCSI): The rCSI module contains questions around the use of the following coping mechanisms in the last seven days: relying on less preferred/less expensive foods, borrowing food, limiting portion sizes, restricting consumption by adults for children to eat, and reducing the number of meals per day.[36] The number of times each coping strategy was utilized in the last seven days was recorded, and a rCSI score was then calculated by adding up the number of days.[36] A binary variable testing rCSI scores of <19 and ≥19 was constructed, corresponding to phase 1/2 (minimal/stressed) versus phase 3/4/5 (crisis/emergency/famine), respectively, based on guidance from the Integrated Food Security Phase Classification (IPC) and as used by Save the Children.[46,47]

Food Consumption Score (FCS): The FCS module contains questions around days of consumption of the following food groups during the last seven days: cereals, legumes, vegetables, fruits, meat and fish, dairy, sugar, oil, and condiments.[38] Food groups were weighted and then summed to generate a FCS score, according to the following formula [38]:

$$\text{FCS} = (\text{days of cereals} * 2) + (\text{days of legumes} * 3) + (\text{days of vegetables}) + (\text{days of fruit}) + (\text{days of meat and fish} * 4) + (\text{days of dairy} * 4) + (\text{days of sugar} * 0.5) + (\text{days of oil} * 0.5) + (\text{days of condiments} * 0)$$

FCS scores were then categorized into acceptable (>35) (corresponding to IPC Phase 1/2 (Minimal/Stressed)) or unacceptable (≤35) (corresponding to IPC Phase 3/4/5 (Crisis/Emergency/Famine)) levels of dietary diversity and food consumption.[38, 47]

Household Hunger Scale (HHS): The HHS module was administered, asking respondents about the following experiences of household hunger in the 30 days preceding the survey: having no food available in the house, any household member going to sleep hungry due to a lack of available food in the house, and any household member going a full 24 hours without eating due to a lack of available food.[39] If respondents reported experiencing any of these events, they were asked to specify if the event occurred rarely, sometimes, or often in the past 30 days.[39] Responses of "never" experiencing the event were coded as 0, "rarely" or "sometimes" were coded as 1, and "often" was coded as 2.[39] These scores for each question were then summed to calculate a final

HHS score.[39] These scores were then further categorized into the following groups: little-to-no hunger (scores of 0-1), moderate hunger (scores of 2-3), and severe hunger (scores of 4-6).[39] For the purposes of this analysis, the moderate and severe hunger categories were collapsed to create a binary indicator comparing moderate-severe household hunger (corresponding to IPC Phase 3/4/5 (Crisis/Emergency/Famine)) versus little-to-no household hunger (corresponding to IPC Phase 1/2 (Minimal/Stressed)).[47]

Household Crowding: A household crowding index was calculated by dividing the number of individuals living in the household by the number of rooms in the household, as done elsewhere.[37] Responses greater than 3 for number of rooms in the household were re-coded as 3, for, given the context, respondents were primarily living in tents with typically a maximum of 3 rooms (for example, a response of 22 rooms was recoded as 3). Crowding index values were then categorized into a binary variable of crowded (score ≥ 5) versus not crowded (score < 5).

Maternal Health-Related Knowledge: A composite health-related knowledge variable was created, combining knowledge around exclusive breastfeeding, timing of breastfeeding initiation, timing of liquids initiation, timing of solid food initiation, handwashing moments, and water treatment methods. For each of these areas, a binary variable was created, coding correct knowledge as 1 and incorrect knowledge as 0. These six binary variables were then summed to arrive at a final composite knowledge score, ranging from 0-6, with 6 reflecting the best health-related knowledge. After examining the distribution of scores, a binary health-related knowledge variable was created to compare "high" knowledge (scores of 5-6) with "low-to-moderate" knowledge (scores of 0-4).

Exclusive breastfeeding knowledge was considered correct if a participant reported that infants should only receive breastmilk and no other liquids or solids for the first 6 months of life, and timing of breastfeeding initiation was correct if the participant identified that breastfeeding should be initiated within one hour of delivery.[48,49] Participants were considered to have correct knowledge on timing of liquids and solids introduction if they reported that introduction should occur when children are 6-8 months old.[49] Finally, correct knowledge of handwashing moments included if participants accurately identified at least 3/5 critical handwashing moments (before preparing food, before eating, before feeding children, after handling a child's stool/diaper, after using the latrine/toilet), and correct knowledge of water treatment methods included if participants identified at least one recommended method for treating water prior to consumption (boiling, chlorinating, keeping under the sun, filtering, using aquatab).[40,50]

Minimum Dietary Diversity for Children (MDD-C): MDD-C was calculated two ways- either including or excluding breastmilk as a food group - based on the age group of children and the relevance of breastmilk as an important dietary component.[27] If breastmilk was included, dietary diversity was considered to be met if infants consumed at least 5/8 food groups in the previous day.[27] If breastmilk was excluded, dietary diversity was met by consuming at least 4/7 food groups in the previous days.[27] While the present study included children aged 9-59 months old, the majority of children were aged 24-59 months (72.8% and 73.3% aged 24-59 months at midline and endline, respectively), with breastfeeding being less applicable for these older ages, as global recommendations encourage breastfeeding from birth to 2+ years, with complementary

foods (starting at 6 months) comprising an increasingly larger share of nutritional requirements as children get older.[48] Therefore, to not underestimate dietary diversity, for the overall CU5 models and the stratified models of children aged 24-59 months, MDD-C was calculated without breastmilk as a food group (referred to in this analysis as MDDC7), as used elsewhere for children over 23 months.[51–53] For the stratified models of children 9-23 months old, MDD-C with breastmilk as a food group was calculated, as breastmilk is a critical food group for this age group (referred to as MDDC8 in this analysis), and this approach is recommended for children aged 6-23 months.[27]

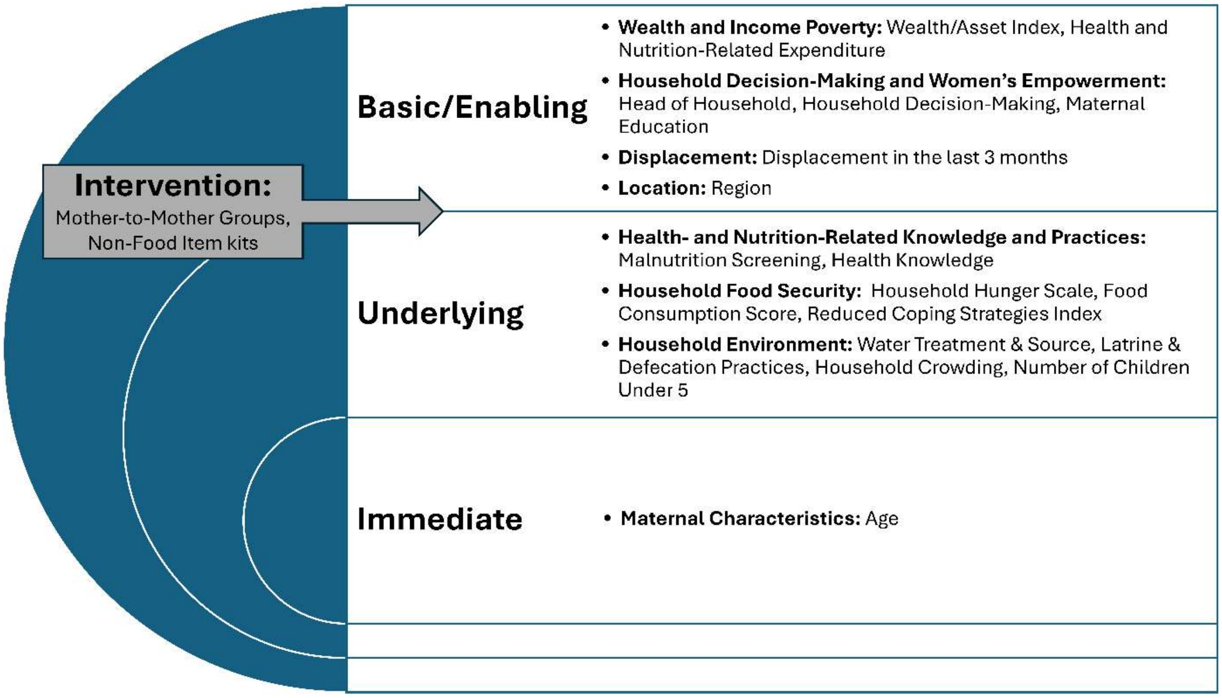

Figure S1. Mapping of Variables Tested in the Mothers' Models

Text S6: Defining the Study Sample

Details of the study population, trial participant selection criteria, and sample size calculation used for the cRCT are provided elsewhere.[11] In the cRCT, data were collected on household conditions/factors, and anthropometric data on all CU5 (aged 6-59 months) in the household were collected; however, child characteristic questions around diet, disease, and vaccination status were only asked of the youngest child in each household.[10,11] Therefore, to be able to study these important factors, the child samples for this analysis focused only on the youngest child in each household aged 6-59 months. In the original data sets, there were 1263 youngest children-caregiver pairs at midline and 1157 youngest child-caregiver pairs at endline. In order to have a clean sample of mother-child pairs to be able to directly study associations between maternal nutrition status and child nutrition status, observations were removed if the reported caregiver was not the child's mother or if anthropometric data was missing. In both the child and mother datasets, mothers with reported ages above 65 were excluded from the analysis, as it was assumed that these women were likely the child's grandmother. In only the samples for the CU5 models, children were excluded if older than 59 months and if the child's WHZ was

outside of the biologically plausible range of -5 to +5 SD. Additionally, children were excluded if the mother had given birth to a newborn since baseline to avoid any contamination around diet and disease questions concerning the youngest child in the household. In only the samples for the mothers' wasting analyses, mothers were excluded from the analysis if they had an extremely high (>60 cm) or low (8 cm) biologically implausible MUAC. This process resulted in final sample sizes of 956 children and 1066 mothers at midline and 833 children and 1023 mothers at endline. See **Figures S2-S5** for the flow diagram of each analysis sample.

Text S7: Modeling Strategy

A backwards elimination model-building approach was implemented in a hierarchical way to assess the correlates of wasting, employing the basic/enabling, intervention, underlying, and immediate levels described in **Figure 1**.[54–56] Complete case analysis was performed. During the first stage of analysis, crude, unadjusted bivariate relationships between the outcome of interest and each independent variable were examined, demonstrating the overall, crude relationship between each variable and the outcome (first column in each correlates results table). Next, major confounder-adjusted relationships were examined, adjusting for child sex and age, household region, and trial arm in the CU5 models and adjusting for household region and trial arm in the mothers' models (second column in each correlates results table). These major confounder-adjusted regression results depict the overall relationship between each variable and the outcome after adjusting for any potential confounding effects of child age, sex, household region, and trial arm.

From the major confounder-adjusted results, any independent variable that had an association with the outcome of interest with a p-value <0.20 was retained for the final model-building approach, as used elsewhere.[56] A backwards elimination model building approach was used and implemented in a hierarchical way at four levels (corresponding to Models 1-4 described below) according to our statistical framework (third column in each correlates results table).[54-56] Any independent variables from the basic/enabling level with a p-value <0.20 from the major confounder-adjusted association with the outcome of interest were entered into an initial regression model that adjusted for child age, sex, household region, and the trial arm in the child models and household region and trial arm in the mother models.[57] Using a backwards elimination approach, variables with the highest p-values were removed one at a time until all remaining independent variables had a p-value ≤0.10. The coefficients, 95% confidence intervals, and p-values for these variables were reported (Model 1: basic/enabling correlates with p≤0.10). Next, now ignoring the p-values of any retained basic/enabling variables, any intervention-related variables that had a p-value <0.20 from the adjusted associations were added to the model, again removing variables one at a time until all (if any) remaining intervention-related variables had a p-value ≤0.10. These coefficients, 95% confidence intervals, and p-values were then reported (Model 2: Model 1 + intervention variables with p≤0.10), if applicable. This process was then repeated with the underlying level variables (Model 3 = Model 2 + underlying variables with p≤0.10) and then finally with the immediate level variables (Model 4 = Model 3 + immediate variables with p≤0.10). Following the methods of Victora, this approach accounts for the hierarchical and interrelated nature of correlates (as characterized by the conceptual framework), allowing for the "true"

association of a variable with the outcome to be reported without confounding by a more distal variable.[56] The results presented in the paper focus on the findings from the fully adjusted models using the hierarchical strategy, as these are believed to represent the most unbiased and unconfounded results for potential correlates by accounting for the interrelated hierarchical nature of the correlates of malnutrition.[54-56]

Throughout all analyses, a statistical significance level of $\alpha = 0.05$ is considered. Variables with a p-value above 0.05 and less than or equal to 0.10 were considered marginally statistically significant. For the purposes of this study, the cRCT trial arm was adjusted for in the analysis to control for any effect of the interventions. To not overfit models, the village cluster was not controlled for in this analysis.

For examining WHZ among CU5, an overall model with the entire sample of children was built with both the midline data and endline data (separately). Additional models were stratified by region, due to disproportionate wasting burdens by region, and by child age to assess correlates among children aged 9-23 months old versus 24-59 months old.

Regarding maternal wasting by MUAC, only overall models with all mothers were built at midline and endline. In contrast to child wasting, there was not a significant difference in the burden of wasting among mothers by region (chi-squared p-value = 0.182 at midline and 0.579 at endline).

For all final multiple linear regression models with the hierarchical approach (model 4 described above), Q-Q plots and Kernel Density Estimate plots of residuals were examined to assess normality of residuals, and residual-versus-fitted (RVF) plots and the Breusch-Pagan/Cook-Weisberg test were assessed to check for homoskedasticity. For any final multiple linear regression models with the hierarchical approach (model 4 described above) that had evidence of heteroskedasticity via the Breusch-Pagan/Cook-Weisberg test, the four-stage model-building approach was repeated, using the “robust” option in STATA for robust standard errors. Additionally, histograms of difference in fits (DFITS) values were checked to identify any potential influential points. For all final multivariable Poisson regression models with the hierarchical approach (model 4 described above), Pearson and Deviance goodness-of-fit tests were performed. Collinearity among independent variables was checked in all final adjusted models with the hierarchical approach (model 4 described above) via assessing variance inflation factors (VIF). A conservative cutoff value of $VIF \geq 2.5$ was considered for potential collinearity.[58]

Appendix S2. Results Appendix

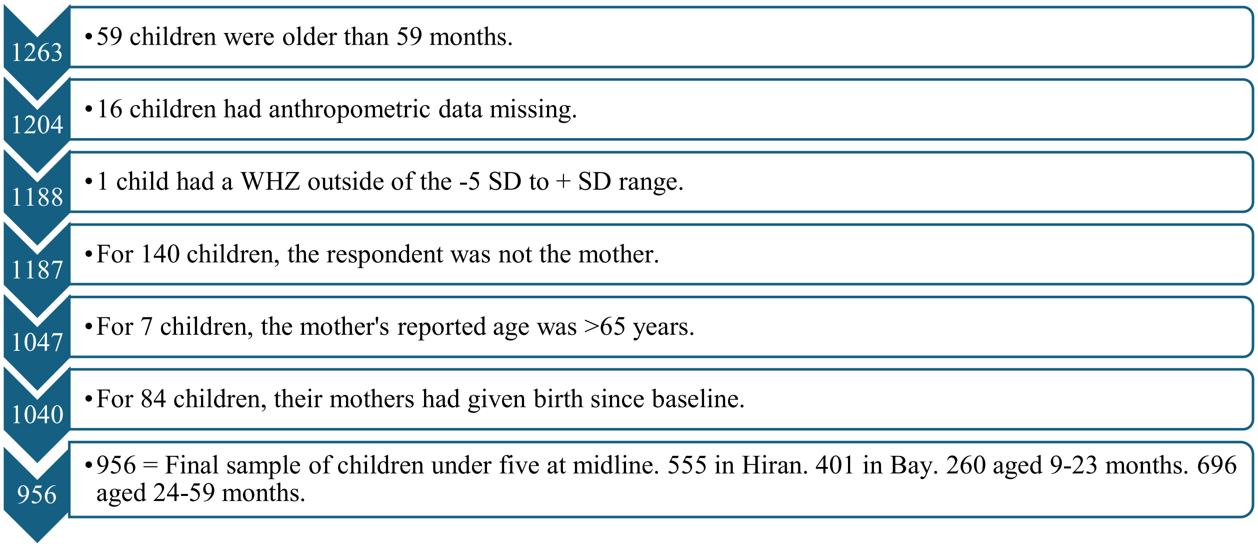

Figure S2. Flow Diagram for the Midline CU5 Sample.

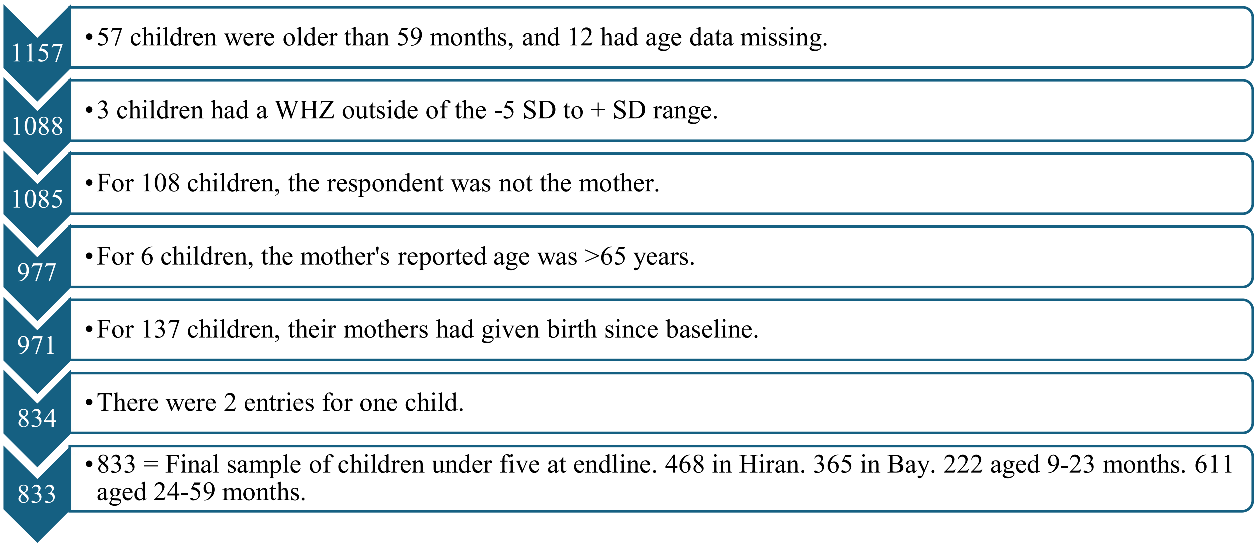

Figure S3. Flow Diagram for the Endline CU5 Sample.

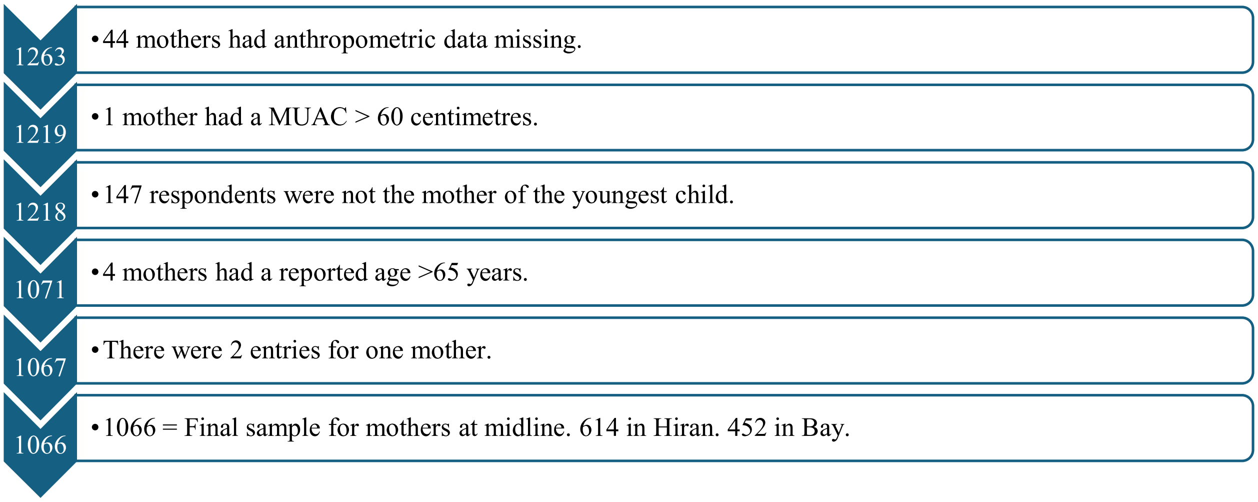

Figure S4. Flow Diagram for the Midline Mother Sample.

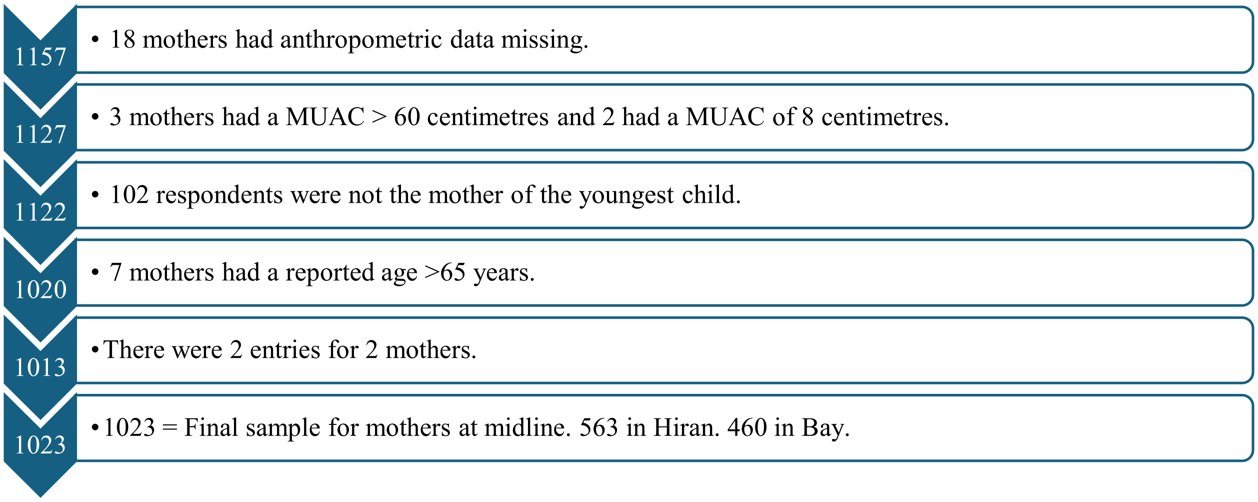

Figure S5. Flow Diagram for the Endline Mother Sample.

Table S3. Additional Child, Mother, and Household Characteristics*

Characteristic*	Midline	Endline
**Immediate Level**		
Child Minimum Dietary Diversity†	n=956	n=833
Acceptable	370 (38.7%)	347 (41.7%)
Unacceptable	586 (61.3%)	486 (58.3%)
Child Egg and/or Flesh-Food Consumption	n=956	n=833
Consumed	420 (43.9%)	361 (43.3%)
Not Consumed	536 (56.1%)	472 (56.7%)
Child Fruit/Vegetable Consumption	n=956	n=833
Consumed	497 (52.0%)	444 (53.3%)

Not Consumed	459 (48.0%)	389 (46.7%)
Child Vitamin A-Rich Food Consumption	n=956	n=833
Consumed	594 (62.1%)	496 (59.5%)
Not Consumed	362 (37.9%)	337 (40.5%)
Child Iron-Rich Food Consumption	n=956	n=833
Consumed	587 (61.4%)	501 (60.1%)
Not Consumed	369 (38.6%)	332 (39.9%)
Child Sugary Food Consumption	n=956	n=833
Consumed	556 (58.2%)	466 (55.9%)
Not Consumed	400 (41.8%)	367 (44.1%)
Maternal Pregnancy Status	n=1066	n=1022
Pregnant	203 (19.0%)	159 (15.6%)
Not Pregnant	863 (81.0%)	863 (84.4%)
**Underlying Level**		
Child Tuberculosis Vaccination	n=956	n=833
Vaccinated	442 (46.2%)	534 (64.1%)
Not Vaccinated	514 (53.8%)	299 (35.9%)
Maternal Health-Related Knowledge	n=1066	n=1023
High	710 (66.6%)	651 (63.6%)
Low-Moderate	356 (33.4%)	372 (36.4%)
Household Number of Children Under 5	n=1066	n=1023
1 Child	794 (74.5%)	764 (74.7%)
2+ Children	272 (25.5%)	259 (25.3%)
Household Crowding	n=1056	--
Crowded	423 (40.1%)	--
Not Crowded	633 (59.9%)	--
Household Reduced Coping Strategy Index	n=1066	n=1023
<19‡	826 (77.5%)	726 (71.0%)
≥19§	240 (22.5%)	297 (29.0%)
Child Malnutrition Screening in Past 3 Months	n=956	n=833
Child Screened	419 (43.8%)	312 (37.5%)
Child Not Screened	537 (56.2%)	521 (62.5%)
Mother Malnutrition Screening in Past 3 Months	n=1066	n=1023
Mother Screened	649 (60.9%)	694 (67.8%)
Mother Not Screened	417 (39.1%)	329 (32.2%)
Household Use of Water Treatment	n=1066	n=1023
Yes	837 (78.5%)	880 (86.0%)
No	229 (21.5%)	143 (14.0%)
Household Disposal of Child's Stool	n=1066	n=1023
Open Area	683 (64.1%)	601 (58.8%)
Latrine/Toilet	383 (35.9%)	422 (41.3%)

Maternal Defecation Practices	n=1066	n=1023
Open Area	325 (30.5%)	429 (41.9%)
Latrine/Toilet	741 (69.5%)	594 (58.1%)
**Intervention Level**		
Mother Attended Mother-to-Mother Support Groups	n=1066	n=1023
Attended	516 (48.4%)	737 (72.0%)
Did Not Attend¶	550 (51.6%)	286 (28.0%)
Received Non-Food Item Kits	n=1066	n=1023
Yes	193 (18.1%)	380 (37.2%)
No	873 (81.9%)	643 (62.9%)
**Basic/Enabling Level**		
Maternal Education	--	n=1022
Some Education	--	314 (30.7%)
No Education	--	708 (69.3%)
Displacement in the Last 3 Months	--	n=1023
Displaced	--	145 (14.2%)
Not Displaced	--	878 (85.8%)
Head of Household	--	n=1022
Mother	--	575 (56.3%)
Father or Other	--	447 (43.7%)
Health- and Nutrition-Related Expenditure	n=1066	n=1023
Lower 25%	265 (24.9%)	255 (24.9%)
Middle 50%	535 (50.2%)	512 (50.1%)
Upper 50%	266 (25.0%)	256 (25.0%)

*Child characteristics are according to child samples at midline and endline. Mother, intervention, and household characteristics are according to mother samples at midline and endline.
†MDDC7.
‡ Corresponding to Integrated Food Security Phase Classification 1/2, i.e. Minimal/Stressed
§ Corresponding to Integrated Food Security Phase Classification 3/4/5, i.e. Crisis/Emergency/Famine.
¶ Among mothers who did not attend M2M groups, some reported that groups were not available in their area while others reported groups were available, but they did not attend.

Table S4. Midline Overall CU5 Model: Correlates of Wasting (n=954)*

	**Outcome: Wasting (Weight-for-Height Z-Score <-2 SD for children under five)**		
	**Unadjusted Bivariate Poisson Regression**	**Major Confounder-Adjusted Poisson Regression†**	**Final Multivariable Poisson Regression (Hierarchical Approach)†**
	Relative Risk (95% CI) p-value	Relative Risk (95% CI) p-value	Relative Risk (95% CI) p-value
**Basic/Enabling Level**			
Wealth/Asset Index (Reference: Not Poor)			
Poor	0.58 (0.40, 0.83) 0.003	1.07 (0.72, 1.59) 0.747	--
Health and Nutrition-Related Expenditure (Reference: Middle 50%)			
Lower 25%	0.84 (0.53, 1.32)	1.00 (0.63, 1.60)	--

	0.449	1.000	
Upper 25%	1.04 (0.68, 1.59)<br>0.848	0.84 (0.55, 1.28)<br>0.410	--
Decision-Making around Income, Purchases, & Healthcare (Reference: Joint)			
Maternal	1.45 (0.93, 2.24)<br>0.099	1.11 (0.71, 1.73)<br>0.659	--
Paternal	1.67 (1.10, 2.54)<br>0.016	1.06 (0.69, 1.63)<br>0.775	--
Region (Reference: Bay)			
Hiran	4.83 (2.86, 8.17)<br>0.000	4.74 (2.78, 8.10)<br>0.000	4.74 (2.78, 8.10)<br>0.000
**Intervention Level**			
Attending M2M Groups (Reference: Attended)			
Did Not Attend	1.35 (0.94, 1.94)<br>0.099	0.88 (0.60, 1.30)<br>0.530	--
NFI Kits (Reference: Received)			
Not Received	*Not assessed in model due to 81.8% of the sample not receiving NFI kits*		
**Underlying Level**			
Household Hunger Scale (Reference: Little-to-No Hunger)‡			
Moderate-to-Severe Hunger§	1.57 (1.09, 2.27)<br>0.016	0.95 (0.65, 1.39)<br>0.789	--
Food Consumption Score (Reference: Acceptable)‡			
Unacceptable§	1.22 (0.84, 1.76)<br>0.295	1.02 (0.70, 1.49)<br>0.910	--
Reduced Coping Strategy Index (Reference: <19)‡			
≥19§	*Not assessed in model due to 77.2% of the sample having a rCSI <19*		
Household Crowding (Reference: Not Crowded)			
Crowded	2.21 (1.54, 3.17)<br>0.000	1.14 (0.76, 1.70)<br>0.527	--
Number of CU5 (Reference: 1 Child)			
2+ Children	0.83 (0.54, 1.25)<br>0.368	0.82 (0.52, 1.30)<br>0.399	--
Water Treatment (Reference: Treats Water)			
Does Not Treat Water	*Not assessed in models due to 80.3% of the sample treating water with an appropriate method*		
Water Source (Reference: Piped Water)			
Not Piped	*Not assessed in model due to 77.6% of the sample not having household piped water*		
Household Defecation (Reference: Latrine/Toilet)			
Open Area	*Not assessed in models due to 76.7% of the sample not practicing open defecation*		
Child Stool Disposal (Reference: Latrine/Toilet)			
Open Area	1.53 (1.03, 2.26)<br>0.036	1.48 (0.99, 2.22)<br>0.055	1.48 (0.99, 2.22)<br>0.055
Maternal Defecation (Reference: Latrine/Toilet)			
Open Area	2.16 (1.52, 3.08)<br>0.000	1.45 (1.00, 2.09)<br>0.051	--
Tuberculosis Vaccination (Reference: Vaccinated)			
Not Vaccinated	2.00 (1.36, 2.94)<br>0.000	1.10 (0.72, 1.69)<br>0.646	--
Malnutrition Screening (Reference: Child Screened)			
Child Not Screened	1.18 (0.82, 1.69)<br>0.373	1.18 (0.81, 1.70)<br>0.390	--

Health-Related Knowledge (Reference: High)			
Low-to-Moderate	1.38 (0.96, 1.98)<br>0.082	1.14 (0.79, 1.64)<br>0.489	--
**Immediate Level**			
Ever Breastfed (Reference: Breastfed)			
Never Breastfed	*Not assessed in models due to 81.5% of the sample having ever been breastfed*		
Minimum Dietary Diversity (Reference: Meets)			
Does Not Meet	1.87 (1.25, 2.82)<br>0.002	0.88 (0.56, 1.38)<br>0.570	--
Fruit/Vegetable Intake the Previous Day (Reference: Consumed)			
Did Not Consume	2.09 (1.44, 3.03)<br>0.000	0.98 (0.64, 1.48)<br>0.907	--
Vitamin A Food Intake the Previous Day (Reference: Consumed)			
Did Not Consume	1.72 (1.21, 2.45)<br>0.003	1.00 (0.69, 1.46)<br>0.980	--
Iron-Rich Food Intake the Previous Day (Reference: Consumed)			
Did Not Consume	1.33 (0.93, 1.90)<br>0.116	0.97 (0.67, 1.39)<br>0.848	--
Eggs/Flesh Food Intake the Previous Day (Reference: Consumed)			
Did Not Consume	1.27 (0.88, 1.82)<br>0.202	0.87 (0.59, 1.26)<br>0.459	--
Sugary Food Intake the Previous Day (Reference: Not Consumed)			
Consumed	0.83 (0.58, 1.19)<br>0.312	1.02 (0.71, 1.46)<br>0.914	--
Illness in the Last 2 Weeks (Reference: No Illness)			
Illness	1.26 (0.87, 1.81)<br>0.220	1.40 (0.97, 2.02)<br>0.072	--
Maternal MUAC (Reference: 24-29.5 cm)			
Underweight (<24 cm)	1.48 (0.96, 2.30)<br>0.077	1.55 (0.99, 2.41)<br>0.053	1.54 (0.99, 2.39)<br>0.057
Overweight (>29.5 cm)	0.66 (0.41, 1.05)<br>0.082	0.72 (0.45, 1.16)<br>0.177	0.77 (0.47, 1.24)<br>0.277
Maternal Age (Reference: 24-34 Years)			
Younger (< 24)	1.13 (0.66, 1.91)<br>0.658	1.31 (0.77, 2.23)<br>0.316	--
Older (35+)	1.20 (0.82, 1.76)<br>0.358	1.16 (0.79, 1.72)<br>0.448	--

CI – confidence interval, cm – centimetres, CU5 – children under five, M2M – mother-to-mother, MUAC – mid-upper arm circumference, NFI – non-food item, SD – standard deviation
*Note that final adjusted models may have a smaller sample size due to a few variables missing some observations. Green indicates statistically significant at $p \leq 0.05$. Orange indicates marginally statistically significant from $0.05 < p \leq 0.10$. Pearson goodness-of-fit p-value for final fully adjusted model = 0.7986.
† Adjusting for child age, sex, household region, and trial arm.
‡ Corresponding to Integrated Food Security Phase Classification 1/2, i.e. Minimal/Stressed.
§ Corresponding to Integrated Food Security Phase Classification 3/4/5, i.e. Crisis/Emergency/Famine.

Table S5. Endline Overall CU5 Model: Correlates of Wasting (n=833)*

	**Outcome: Wasting (Weight-for-Height Z-Score <-2 SD for children under five)**		
	**Unadjusted Bivariate Poisson Regression**	**Major Confounder-Adjusted Poisson Regression†**	**Final Multivariable Poisson Regression (Hierarchical Approach) †**
	Relative Risk (95% CI)	Relative Risk (95% CI)	Relative Risk (95% CI)

	p-value	p-value	p-value
**Basic/Enabling Level**			
Wealth/Asset Index (Reference: Not Poor)			
Poor	0.39 (0.25, 0.61)<br>0.000	0.75 (0.41, 1.37)<br>0.350	--
Health and Nutrition-Related Expenditure (Reference: Middle 50%)			
Lower 25%	0.98 (0.63, 1.52)<br>0.914	1.08 (0.69, 1.70)<br>0.725	--
Upper 25%	1.06 (0.69, 1.63)<br>0.797	0.85 (0.55, 1.32)<br>0.473	--
Head of Household (Reference: Mother)			
Father or Other	0.60 (0.41, 0.89)<br>0.012	0.75 (0.51, 1.12)<br>0.165	--
Decision-Making around Income, Purchases, & Healthcare (Reference: Joint)			
Maternal	1.72 (1.15, 2.57)<br>0.009	1.41 (0.93, 2.12)<br>0.102	--
Paternal	1.41 (0.84, 2.35)<br>0.192	1.14 (0.68, 1.91)<br>0.628	--
Maternal Education (Reference: Some Education)			
No Education	1.05 (0.71, 1.56)<br>0.789	1.19 (0.80, 1.76)<br>0.399	--
Displacement in the Last 3 Months (Reference: Not Displaced)			
Displaced	*Not assessed due to 86.1% of the sample not being displaced*		
Region (Reference: Bay)			
Hiran	2.82 (1.83, 4.35)<br>0.000	2.81 (1.80, 4.39)<br>0.000	2.81 (1.80, 4.39)<br>0.000
**Intervention Level**			
Attending M2M Groups (Reference: Attended)			
Did Not Attend	0.78 (0.51, 1.19)<br>0.247	0.68 (0.44, 1.05)<br>0.085	0.68 (0.44, 1.05)<br>0.085
NFI Kits (Reference: Received)			
Not Received	0.61 (0.43, 0.88)<br>0.008	0.79 (0.55, 1.15)<br>0.226	--
**Underlying Level**			
Household Hunger Scale (Reference: Little-to-no Hunger)‡			
Moderate-to-Severe Hunger§	1.65 (1.14, 2.39)<br>0.008	1.20 (0.82, 1.78)<br>0.348	--
Food Consumption Score (Reference: Acceptable)‡			
Unacceptable§	1.23 (0.86, 1.75)<br>0.266	1.02 (0.71, 1.48)<br>0.897	--
Reduced Coping Strategy Index (Reference: <19)‡			
≥19§	1.43 (0.99, 2.06)<br>0.059	1.27 (0.88, 1.85)<br>0.200	--
Number of CU5 (Reference: 1 Child)			
2+ Children	*Not assessed due to 75.2% of the sample having one child-under-5 in the home*		
Water Treatment (Reference: Treats Water)			
Does Not Treat Water	*Not assessed due to 88.1% of the sample treating water*		
Water Source (Reference: Piped Water)			
Not Piped	1.14 (0.78, 1.67)<br>0.505	0.96 (0.65, 1.42)<br>0.828	--
Household Defecation (Reference: Latrine/Toilet)			
Open Area	*Not assessed due to 76.1% of the sample not practicing open defecation*		
Child Stool Disposal (Reference: Latrine/Toilet)			
Open Area	1.69 (1.14, 2.50)	1.50 (1.01, 2.22)	1.53 (1.03, 2.27)

	0.008	0.045	0.034
Maternal Defecation (Reference: Latrine/Toilet)			
Open Area	1.99 (1.38, 2.87)<br>0.000	1.48 (1.01, 2.16)<br>0.043	--
Tuberculosis Vaccination (Reference: Vaccinated)			
Not Vaccinated	1.56 (1.09, 2.24)<br>0.015	1.06 (0.72, 1.55)<br>0.773	--
Malnutrition Screening (Reference: Child Screened)			
Child Not Screened	1.58 (1.06, 2.36)<br>0.026	1.37 (0.91, 2.05)<br>0.130	
Health-Related Knowledge (Reference: High)			
Low-to-Moderate	1.25 (0.87, 1.80)<br>0.226	0.99 (0.68, 1.45)<br>0.970	--
**Immediate Level**			
Ever Breastfed (Reference: Breastfed)			
Never Breastfed	*Not assessed due to 89.6% of the sample ever being breastfed*		
Minimum Dietary Diversity (Reference: Meets)			
Does Not Meet	1.54 (1.05, 2.26)<br>0.028	0.87 (0.55, 1.38)<br>0.550	--
Fruit/Vegetable Intake the Previous Day (Reference: Consumed)			
Did Not Consume	1.30 (0.91, 1.87)<br>0.146	0.82 (0.55, 1.24)<br>0.357	--
Vitamin A Food Intake the Previous Day (Reference: Consumed)			
Did Not Consume	1.52 (1.06, 2.18)<br>0.021	1.13 (0.77, 1.67)<br>0.531	--
Iron-Rich Food Intake the Previous Day (Reference: Consumed)			
Did Not Consume	1.23 (0.86, 1.77)<br>0.251	0.93 (0.64, 1.36)<br>0.707	--
Eggs/Flesh Food Intake the Previous Day (Reference: Consumed)			
Did Not Consume	1.86 (1.25, 2.75)<br>0.002	1.25 (0.80, 1.97)<br>0.332	--
Sugary Food Intake the Previous Day (Reference: Not Consumed)			
Consumed	0.56 (0.39, 0.81)<br>0.002	0.63 (0.44, 0.92)<br>0.017	0.67 (0.46, 0.98)<br>0.040
Illness in the Last 2 Weeks (Reference: No Illness)			
Illness	1.23 (0.85, 1.80)<br>0.271	1.22 (0.84, 1.79)<br>0.293	--
Maternal MUAC (Reference: 24-29.5 cm)			
Underweight (<24 cm)	1.11 (0.71, 1.74)<br>0.634	1.13 (0.72, 1.77)<br>0.596	--
Overweight (>29.5 cm)	0.90 (0.58, 1.39)<br>0.630	0.93 (0.60, 1.45)<br>0.743	--
Maternal Age (Reference: 24-34 Years)			
Younger (< 24)	1.12 (0.65, 1.93)<br>0.674	1.31 (0.76, 2.26)<br>0.329	--
Older (35+)	1.22 (0.83, 1.80)<br>0.320	1.20 (0.81, 1.79)<br>0.358	--

CI – confidence interval, cm – centimetres, CU5 – children under five, M2M – mother-to-mother, MUAC – mid-upper arm circumference, NFI – non-food item, SD – standard deviation
*Note that final adjusted models may have a smaller sample size due to a few variables missing some observations. Green indicates statistically significant at $p \leq 0.05$. Orange indicates marginally statistically significant from $0.05 < p \leq 0.10$. Pearson goodness-of-fit p-value for final fully adjusted model = 0.9974.
† Adjusting for child age, sex, household region, and trial arm.
‡ Corresponding to Integrated Food Security Phase Classification 1/2, i.e. Minimal/Stressed.

§ Corresponding to Integrated Food Security Phase Classification 3/4/5, i.e. Crisis/Emergency/Famine.

Table S6. Midline 9-23 Months Model: Correlates of Weight-for-Height Z-Score (n=260)*

	**Outcome: WHZ (Weight-for-Height Z-Score for children under five)**		
	**Unadjusted Bivariate Linear Regression**	**Major Confounder-Adjusted Linear Regression†**	**Final Multivariable Linear Regression (Hierarchical Approach)†**
	Coefficient (95% CI) p-value	Coefficient (95% CI) p-value	Coefficient (95% CI) p-value
**Basic/Enabling Level**			
Wealth/Asset Index (Reference: Not Poor)			
Poor	-0.10 (-0.38, 0.18) 0.482	-0.20 (-0.53, 0.13) 0.224	--
Health and Nutrition-Related Expenditure (Reference: Middle 50%)			
Lower 25%	-0.04 (-0.38, 0.30) 0.804	0.01 (-0.35, 0.36) 0.972	--
Upper 25%	0.06 (-0.31, 0.42) 0.760	0.15 (-0.22, 0.51) 0.431	--
Decision-Making around Income, Purchases, & Healthcare (Reference: Joint)			
Maternal	0.15 (-0.23, 0.53) 0.444	0.18 (-0.21, 0.56) 0.370	--
Paternal	-0.07 (-0.42, 0.29) 0.721	0.03 (-0.35, 0.41) 0.890	--
Region (Reference: Bay)			
Hiran	-0.13 (-0.42, 0.15) 0.346	-0.26 (-0.55, 0.02) 0.073	-0.26 (-0.55, 0.02) 0.073
**Intervention Level**			
Attending M2M Groups (Reference: Attended)			
Did Not Attend	-0.01 (-0.29, 0.27) 0.953	0.23 (-0.07, 0.53) 0.139	--
NFI Kits (Reference: Received)			
Not Received	*Not assessed due to 82.3% of the sample not receiving NFI kits*		
**Underlying Level**			
Household Hunger Scale (Reference: Little-to-No hunger) ‡			
Moderate-to-Severe Hunger§	*Not assessed due to 77.7% of the sample having little-to-no household hunger*		
Food Consumption Score (Reference: Acceptable)‡			
Unacceptable§	-0.22 (-0.51, 0.08) 0.147	-0.14 (-0.44, 0.15) 0.344	--
Reduced Coping Strategy Index (Reference: <19)‡			
≥19§	0.20 (-0.12, 0.52) 0.214	0.15 (-0.19, 0.49) 0.401	--
Household Crowding (Reference: Not Crowded)			
Crowded	0.05 (-0.24, 0.34) 0.745	0.24 (-0.14, 0.61) 0.214	--
Number of CU5 (Reference: 1 Child)			
2+ Children	0.36 (0.08, 0.64) 0.011	0.36 (0.07, 0.64) 0.014	0.34 (0.06, 0.63) 0.019
Water Treatment (Reference: Treats Water)			
Does Not Treat Water	*Not assessed due to 81.5% of the sample practicing water treatment by an appropriate method*		
Water Source (Reference: Piped Water)			

Not Piped	0.04 (-0.28, 0.36)<br>0.803	0.04 (-0.28, 0.36)<br>0.814	--
Household Defecation (Reference: Latrine/Toilet)			
Open Area	*Not assessed due to 82.3% of the sample not practicing open defecation*		
Child Stool Disposal (Reference: Latrine/Toilet)			
Open Area	-0.29 (-0.58, -0.00)<br>0.049	-0.22 (-0.51, 0.07)<br>0.137	--
Maternal Defecation (Reference: Latrine/Toilet)			
Open Area	-0.25 (-0.56, 0.07)<br>0.132	-0.13 (-0.46, 0.20)<br>0.440	--
Tuberculosis Vaccination (Reference: Vaccinated)			
Not Vaccinated	-0.30 (-0.58, -0.02)<br>0.036	-0.29 (-0.60, 0.02)<br>0.069	-0.26 (-0.57, 0.05)<br>0.096
Malnutrition Screening (Reference: Child Screened)			
Child Not Screened	-0.04 (-0.32, 0.25)<br>0.806	-0.05 (-0.34, 0.23)<br>0.707	
Health and Nutrition Knowledge (Reference: High)			
Low-to-Moderate	-0.02 (-0.32, 0.28)<br>0.890	0.04 (-0.25, 0.34)<br>0.772	--
**Immediate Level**			
Ever Breastfed (Reference: Breastfed)			
Never Breastfed	*Not assessed due to 86.5% of the sample having ever been breastfed*		
Minimum Dietary Diversity (Reference: Meets)			
Does Not Meet	-0.10 (-0.41, 0.21)<br>0.516	-0.05 (-0.39, 0.29)<br>0.771	--
Fruit/Vegetable Intake the Previous Day (Reference: Consumed)			
Did Not Consume	-0.01 (-0.29, 0.27)<br>0.950	0.15 (-0.17, 0.48)<br>0.352	--
Vitamin A Food Intake the Previous Day (Reference: Consumed)			
Did Not Consume	-0.07 (-0.35, 0.22)<br>0.638	0.07 (-0.25, 0.39)<br>0.661	--
Iron-Rich Food Intake the Previous Day (Reference: Consumed)			
Did Not Consume	-0.01 (-0.29, 0.27)<br>0.950	0.02 (-0.28, 0.32)<br>0.884	--
Egg/Flesh Food Intake the Previous Day (Reference: Consumed)			
Did Not Consume	-0.11 (-0.39, 0.18)<br>0.469	-0.05 (-0.35, 0.25)<br>0.745	--
Sugary Food Intake the Previous Day (Reference: Not Consumed)			
Consumed	0.04 (-0.24, 0.33)<br>0.757	0.05 (-0.24, 0.33)<br>0.748	--
Illness in the Last 2 Weeks (Reference: No Illness)			
Illness	-0.04 (-0.34, 0.26)<br>0.791	-0.02 (-0.32, 0.27)<br>0.877	--
Maternal MUAC (Reference: 24-29.5 cm)			
Underweight (<24 cm)	-0.35 (-0.75, 0.05)<br>0.083	-0.31 (-0.71, 0.09)<br>0.124	--
Overweight (>29.5 cm)	0.09 (-0.23, 0.41)<br>0.585	0.06 (-0.26, 0.37)<br>0.717	--
Maternal Age (Reference: 24-34 Years)			
Younger (<24)	-0.11 (-0.49, 0.26)<br>0.544	-0.14 (-0.51, 0.23)<br>0.449	--
Older (35+)	0.19 (-0.12, 0.51)<br>0.229	0.17 (-0.14, 0.49)<br>0.275	--

CI – confidence interval, cm – centimetres, CU5 – children under five, M2M – mother-to-mother, MUAC – mid-upper arm circumference, NFI – non-food item
*Note that final adjusted models may have a smaller sample size due to a few variables missing some observations. Green indicates statistically significant at $p \leq 0.05$. Orange indicates marginally statistically significant from $0.05 < p \leq 0.10$.
† Adjusting for child age, sex, household region, and trial arm.
‡ Corresponding to Integrated Food Security Phase Classification 1/2, i.e. Minimal/Stressed.
§ Corresponding to Integrated Food Security Phase Classification 3/4/5, i.e. Crisis/Emergency/Famine.

Table S7. Midline 24-59 Months Model: Correlates of Weight-for-Height Z-Score (n=696)*

	**Outcome: WHZ (Weight-for-Height Z-Score for children under five)**		
	**Unadjusted Bivariate Linear Regression**	**Major Confounder-Adjusted Linear Regression†**	**Final Multivariable Linear Regression (Hierarchical Approach)†**
	Coefficient (95% CI) p-value	Coefficient (95% CI) p-value	Coefficient (95% CI) p-value
**Basic/Enabling Level**			
Wealth/Asset Index (Reference: Not Poor)			
Poor	0.49 (0.32, 0.65) 0.000	0.07 (-0.11, 0.25) 0.434	--
Health and Nutrition-Related Expenditure (Reference: Middle 50%):			
Lower 25%	0.16 (-0.05, 0.37) 0.143	0.01 (-0.19, 0.21) 0.927	--
Upper 25%	-0.09 (-0.30, 0.11) 0.360	0.03 (-0.15, 0.22) 0.716	--
Decision-Making around Income, Purchases, & Healthcare (Reference: Joint)			
Maternal	-0.33 (-0.54, -0.12) 0.002	-0.16 (-0.35, 0.03) 0.098	-0.16 (-0.35, 0.03) 0.098
Paternal	-0.38 (-0.59, -0.17) 0.000	-0.09 (-0.28, 0.11) 0.386	-0.09 (-0.28, 0.11) 0.386
Region (Reference: Bay)			
Hiran	-0.88 (-1.04, -0.73) 0.000	-0.82 (-0.98, -0.66) 0.000	-0.80 (-0.97, -0.64) 0.000
**Intervention Level**			
Attending M2M Groups (Reference: Attended)			
Did Not Attend	-0.20 (-0.37, -0.04) 0.018	0.02 (-0.15, 0.19) 0.820	--
NFI Kits (Reference: Received)			
Not Received	*Not assessed due to 81.6% of the sample not receiving NFI kits*		
**Underlying Level**			
Household Hunger Scale (Reference: Little-to-No Hunger)‡			
Moderate-to-Severe Hunger§	-0.41 (-0.60, -0.23) 0.000	-0.00 (-0.19, 0.18) 0.962	--
Food Consumption Score (Reference: Acceptable)‡			
Unacceptable§	-0.10 (-0.28, 0.09) 0.309	-0.02 (-0.19, 0.15) 0.824	--
Reduced Coping Strategy Index (Reference: <19)‡			
≥19§	*Not assessed due to 78.3% of the sample having a rCSI <19*		
Household Crowding (Reference: Not Crowded)			
Crowded	-0.56 (-0.73, -0.39) 0.000	-0.07 (-0.26, 0.13) 0.483	--
Number of CU5 (Reference: 1 Child)			

2+ Children	*Not assessed due to 79.3% of the sample having one child-under-5 in the household*		
Water Treatment (Reference: Treats Water)			
Does Not Treat Water	*Not assessed due to 79.9% of the sample treating water with an appropriate method*		
Water Source (Reference: Piped Water)			
Not Piped	*Not assessed due to 78.7% of the sample not having household piped water*		
Household Defecation (Reference: Latrine/Toilet)			
Open Area	-0.58 (-0.77, -0.39)<br>0.000	-0.29 (-0.48, -0.10)<br>0.002	--
Child Stool Disposal (Reference: Latrine/Toilet)			
Open Area	-0.17 (-0.35, 0.00)<br>0.054	-0.20 (-0.36, -0.04)<br>0.016	--
Maternal Defecation (Reference: Latrine/Toilet)			
Open Area	-0.52 (-0.69, -0.34)<br>0.000	-0.28 (-0.45, -0.11)<br>0.001	-0.29 (-0.46, -0.11)<br>0.001
Tuberculosis Vaccination (Reference: Vaccinated)			
Not Vaccinated	-0.44 (-0.61, -0.27)<br>0.000	0.00 (-0.18, 0.18)<br>0.994	--
Malnutrition Screening (Reference: Child Screened)			
Child Not Screened	0.02 (-0.15, 0.19)<br>0.847	0.01 (-0.15, 0.16)<br>0.949	
Health-Related Knowledge (Reference: High)			
Low-to-Moderate	-0.17 (-0.35, 0.01)<br>0.065	0.04 (-0.13, 0.21)<br>0.674	--
**Immediate Level**			
Ever Breastfed (Reference: Breastfed)			
Never Breastfed	*Not assessed due to 79.6% of the sample having ever breastfed*		
Minimum Dietary Diversity (Reference: Meets)			
Does Not Meet	-0.59 (-0.75, -0.42)<br>0.000	-0.13 (-0.31, 0.06)<br>0.183	--
Fruit/Vegetable Intake the Previous Day (Reference: Consumed)			
Did Not Consume	-0.58 (-0.75, -0.42)<br>0.000	-0.06 (-0.25, 0.14)<br>0.554	--
Vitamin A Food Intake the Previous Day (Reference: Consumed)			
Did Not Consume	-0.44 (-0.61, -0.26)<br>0.000	-0.02 (-0.20, 0.16)<br>0.823	--
Iron-Rich Food Intake the Previous Day (Reference: Consumed)			
Did Not Consume	-0.37 (-0.54, -0.19)<br>0.000	-0.12 (-0.28, 0.05)<br>0.160	--
Egg/Flesh Food Intake the Previous Day (Reference: Consumed)			
Did Not Consume	-0.31 (-0.48, -0.14)<br>0.000	-0.06 (-0.22, 0.10)<br>0.451	--
Sugary Food Intake the Previous Day (Reference: Not Consumed)			
Consumed	0.17 (-0.00, 0.34)<br>0.056	-0.03 (-0.19, 0.13)<br>0.711	--
Illness in the Last 2 Weeks (Reference: No Illness)			
Illness	-0.13 (-0.31, 0.05)<br>0.150	-0.17 (-0.33, -0.00)<br>0.045	-0.14 (-0.31, 0.03)<br>0.098
Maternal MUAC (Reference: 24-29.5 cm)			
Underweight (<24 cm)	-0.20 (-0.45, 0.05)<br>0.111	-0.19 (-0.42, 0.03)<br>0.088	-0.20 (-0.42, 0.02)<br>0.079
Overweight (>29.5 cm)	0.20 (0.00, 0.40)<br>0.046	0.18 (-0.00, 0.36)<br>0.052	0.14 (-0.04, 0.32)<br>0.123

Maternal Age (Reference: 24-34)			
Younger (<24)	0.00 (-0.26, 0.27)<br>0.986	-0.02 (-0.26, 0.22)<br>0.872	--
Older (35+)	-0.04 (-0.22, 0.14)<br>0.654	0.01 (-0.15, 0.18)<br>0.860	--

CI – confidence interval, cm – centimetres, CU5 – children under five, M2M – mother-to-mother, MUAC – mid-upper arm circumference, NFI – non-food item

*Note that final adjusted models may have a smaller sample size due to a few variables missing some observations. Green indicates statistically significant at p ≤ 0.05. Orange indicates marginally statistically significant from 0.05 < p ≤ 0.10.

† Adjusting for child age, sex, household region, and trial arm.

‡ Corresponding to Integrated Food Security Phase Classification 1/2, i.e. Minimal/Stressed.

§ Corresponding to Integrated Food Security Phase Classification 3/4/5, i.e. Crisis/Emergency/Famine.

Table S8. Endline 9-23 Month CU5 Model: Correlates of Weight-for-Height Z-Score (n=222)*

	**Outcome: WHZ<br>(Weight-for-Height Z-Score for children under five)**		
	**Unadjusted Bivariate Linear Regression**	**Major Confounder-Adjusted Linear Regression†**	**Final Multivariable Linear Regression (Hierarchical Approach)†**
	Coefficient (95% CI)<br>p-value	Coefficient (95% CI)<br>p-value	Coefficient (95% CI)<br>p-value
**Basic/Enabling Level**			
Wealth/Asset Index (Reference: Not Poor)			
Poor	0.08 (-0.23, 0.40)<br>0.600	-0.00 (-0.45, 0.44)<br>0.991	
Health and Nutrition-Related Expenditure (Reference: Middle 50%)			
Lower 25%	-0.09 (-0.47, 0.30)<br>0.664	-0.17 (-0.56, 0.21)<br>0.378	--
Upper 25%	0.15 (-0.23, 0.53)<br>0.428	0.22 (-0.16, 0.60)<br>0.248	--
Head of Household (Reference: Mother)			
Father or Other	-0.13 (-0.44, 0.19)<br>0.429	-0.15 (-0.47, 0.16)<br>0.345	--
Decision-Making around Income, Purchases, & Healthcare (Reference: Joint)			
Maternal	-0.04 (-0.44, 0.36)<br>0.845	-0.05 (-0.47, 0.37)<br>0.809	--
Paternal	0.42 (-0.07, 0.92)<br>0.092	0.41 (-0.09, 0.90)<br>0.104	--
Maternal Education (Reference: Some Education)			
No Education	-0.54 (-0.87, -0.21)<br>0.001	-0.59 (-0.92, -0.26)<br>0.001	-0.59 (-0.93, -0.25)<br>0.001
Displacement in the Last 3 Months (Reference: Not Displaced)			
Displaced	*Not assessed due to 83.3% of the sample not being displaced in the last 3 months*		
Region (Reference: Bay)			
Hiran	-0.10 (-0.41, 0.22)<br>0.542	-0.24 (-0.57, 0.08)<br>0.146	-0.31 (-0.61, 0.00)<br>0.050
**Intervention Level**			
Attending M2M Groups (Reference: Attended)			
Did Not Attend	0.11 (-0.25, 0.46)<br>0.553	0.24 (-0.12, 0.61)<br>0.193	--
NFI Kits (Reference: Received)			
Not Received	-0.12 (-0.44, 0.20)	-0.05 (-0.38, 0.27)	--

	0.455	0.749	
**Underlying Level**			
Household Hunger Scale (Reference: Little-to-No Hunger)‡			
Moderate-to-Severe Hunger§	0.20 (-0.12, 0.51)<br>0.217	0.23 (-0.10, 0.56)<br>0.171	--
Food Consumption Score (Reference: Acceptable)‡			
Unacceptable§	-0.21 (-0.52, 0.11)<br>0.195	-0.24 (-0.57, 0.08)<br>0.142	--
Reduced Coping Strategy Index (Reference: <19)‡			
≥19§	-0.21 (-0.55, 0.14)<br>0.245	-0.22 (-0.57, 0.12)<br>0.209	--
Number of CU5 (Reference: 1 Child)			
2+ Children	0.12 (-0.19, 0.44)<br>0.450	0.13 (-0.20, 0.46)<br>0.440	--
Water Treatment (Reference: Treats Water)			
Does Not Treat Water	*Not assessed due to 86.9% of the sample treating water*		
Water Source (Reference: Piped Water)			
Not Piped	0.03 (-0.30, 0.36)<br>0.844	0.10 (-0.23, 0.44)<br>0.542	--
Household Defecation (Reference: Latrine/Toilet)			
Open Area	*Not assessed due to 79.7% of the sample not practicing open defecation*		
Child Stool Disposal (Reference: Latrine/Toilet)			
Open Area	-0.21 (-0.52, 0.10)<br>0.188	-0.23 (-0.54, 0.08)<br>0.149	--
Maternal Defecation (Reference: Latrine/Toilet)			
Open Area	-0.34 (-0.66, -0.02)<br>0.035	-0.33 (-0.66, -0.00)<br>0.050	-0.26 (-0.57, 0.05)<br>0.094
Tuberculosis Vaccination (Reference: Vaccinated)			
Not Vaccinated	-0.28 (-0.62, 0.07)<br>0.115	-0.21 (-0.58, 0.16)<br>0.271	--
Malnutrition Screening (Reference: Child Screened)			
Child Not Screened	-0.13 (-0.45, 0.20)<br>0.445	-0.16 (-0.49, 0.16)<br>0.320	--
Health-Related Knowledge (Reference: High)			
Low-to-Moderate	0.01 (-0.32, 0.33)<br>0.964	0.07 (-0.27, 0.41)<br>0.681	--
**Immediate Level**			
Ever Breastfed (Reference: Breastfed)			
Never Breastfed	*Not assessed due to 86.9% of the sample having been ever breastfed*		
Minimum Dietary Diversity** (Reference: Meets)			
Does Not Meet	-0.21 (-0.55, 0.12)<br>0.206	-0.30 (-0.71, 0.10)<br>0.140	--
Fruit/Vegetable Intake the Previous Day (Reference: Consumed)			
Did Not Consume	-0.15 (-0.46, 0.17)<br>0.355	-0.18 (-0.60, 0.23)<br>0.385	--
Vitamin A Food Intake the Previous Day (Reference: Consumed)			
Did Not Consume	-0.17 (-0.49, 0.14)<br>0.286	-0.22 (-0.57, 0.14)<br>0.229	--
Iron-Rich Food Intake the Previous Day (Reference: Consumed)			
Did Not Consume	0.04 (-0.27, 0.36)<br>0.785	0.07 (-0.27, 0.41)<br>0.673	--
Egg/Flesh Food Intake the Previous Day (Reference: Consumed)			
Did Not Consume	-0.17 (-0.48, 0.14)<br>0.285	-0.23 (-0.58, 0.13)<br>0.210	--

Sugary Food Intake the Previous Day (Reference: Not Consumed)			
Consumed	-0.11 (-0.42, 0.21)<br>0.502	-0.05 (-0.38, 0.28)<br>0.766	--
Illness in the Last 2 Weeks (Reference: No Illness)			
Illness	-0.25 (-0.59, 0.09)<br>0.156	-0.25 (-0.59, 0.10)<br>0.157	--
Maternal MUAC (Reference: 24-29.5 cm)			
Underweight (<24 cm)	0.23 (-0.16, 0.62)<br>0.240	0.23 (-0.16, 0.63)<br>0.247	0.22 (-0.16, 0.61)<br>0.260
Overweight (>29.5 cm)	0.63 (0.26, 1.00)<br>0.001	0.57 (0.20, 0.94)<br>0.003	0.48 (0.10, 0.87)<br>0.015
Maternal Age (Reference: 24-34 Years)			
Younger (<24)	-0.05 (-0.46, 0.37)<br>0.826	-0.13 (-0.56, 0.29)<br>0.534	--
Older (35+)	-0.05 (-0.41, 0.31)<br>0.789	-0.11 (-0.47, 0.25)<br>0.544	--

CI – confidence interval, cm – centimetres, CU5 – children under five, M2M – mother-to-mother, MUAC – mid-upper arm circumference, NFI – non-food item

*Note that final adjusted models may have a smaller sample size due to a few variables missing some observations. Green indicates statistically significant at $p \leq 0.05$. Orange indicates marginally statistically significant from $0.05 < p \leq 0.10$.

† Adjusting for child age, sex, household region, and trial arm.

‡ Corresponding to Integrated Food Security Phase Classification 1/2, i.e. Minimal/Stressed.

§ Corresponding to Integrated Food Security Phase Classification 3/4/5, i.e. Crisis/Emergency/Famine.

Table S9. Endline 24-59 Month CU5 Model: Correlates of Weight-for-Height Z-Score (n=611)*

	**Outcome: WHZ (Weight-for-Height Z-Score for children under five)**		
	**Unadjusted Bivariate Linear Regression**	**Major Confounder-Adjusted Linear Regression†**	**Final Multivariable Linear Regression (Hierarchical Approach)†**
	Coefficient (95% CI)<br>p-value	Coefficient (95% CI)<br>p-value	Coefficient (95% CI)<br>p-value
**Basic/Enabling Level**			
Wealth/Asset Index (Reference: Not Poor)			
Poor	0.46 (0.29, 0.63)<br>0.000	-0.13 (-0.37, 0.10)<br>0.265	
Health and Nutrition-Related Expenditure (Reference: Middle 50%)			
Lower 25%	-0.04 (-0.25, 0.17)<br>0.707	-0.14 (-0.33, 0.06)<br>0.170	--
Upper 25%	-0.03 (-0.24, 0.19)<br>0.811	0.13 (-0.08, 0.33)<br>0.224	--
Head of Household (Reference: Mother)			
Father or Other	0.17 (-0.01, 0.34)<br>0.059	-0.04 (-0.21, 0.13)<br>0.626	--
Decision-Making around Income, Purchases, & Healthcare (Reference: Joint)			
Maternal	-0.30 (-0.51, -0.10)<br>0.004	-0.17 (-0.36, 0.03)<br>0.096	-0.17 (-0.36, 0.03)<br>0.096
Paternal	-0.37 (-0.62, -0.12)<br>0.003	-0.20 (-0.44, 0.04)<br>0.095	-0.20 (-0.44, 0.04)<br>0.095
Maternal Education (Reference: Some Education)			
No Education	0.07 (-0.12, 0.26)<br>0.456	-0.06 (-0.24, 0.11)<br>0.493	--

Displacement in the Last 3 Months (Reference: Not Displaced)			
Displaced	*Not assessed due to 87.1% of the sample not being displaced in the last 3 months*		
Region (Reference: Bay)			
Hiran	-0.69 (-0.86, -0.53)<br>0.000	-0.65 (-0.82, -0.48)<br>0.000	-0.62 (-0.79, -0.45)<br>0.000
**Intervention Level**			
Attending M2M Groups (Reference: Attended)			
Did Not Attend	0.14 (-0.05, 0.33)<br>0.162	0.14 (-0.05, 0.32)<br>0.148	--
NFI Kits (Reference: Received)			
Not Received	0.26 (0.09, 0.43)<br>0.003	0.05 (-0.12, 0.22)<br>0.580	--
**Underlying Level**			
Household Hunger Scale (Reference: Little-to-No Hunger)‡			
Moderate-to-Severe Hunger§	-0.28 (-0.45, -0.11)<br>0.001	-0.01 (-0.18, 0.17)<br>0.936	--
Food Consumption Score (Reference: Acceptable)‡			
Unacceptable§	-0.19 (-0.37, -0.02)<br>0.026	-0.10 (-0.27, 0.06)<br>0.205	--
Reduced Coping Strategy Index (Reference: <19)‡			
≥19§	-0.18 (-0.37, 0.00)<br>0.054	-0.08 (-0.25, 0.10)<br>0.377	--
Number of CU5 (Reference: 1 Child)			
2+ Children	*Not assessed due to 82.0% of the sample only having 1 CU5 in the household*		
Water Treatment (Reference: Treats Water)			
Does Not Treat Water	*Not assessed due to 88.5% of the sample treating water*		
Water Source (Reference: Piped Water)			
Not Piped	-0.13 (-0.31, 0.05)<br>0.157	-0.02 (-0.19, 0.15)<br>0.819	--
Household Defecation (Reference: Latrine/Toilet)			
Open Area	-0.04 (-0.24, 0.15)<br>0.657	-0.00 (-0.19, 0.18)<br>0.963	--
Child Stool Disposal (Reference: Latrine/Toilet)			
Open Area	-0.15 (-0.32, 0.03)<br>0.099	-0.10 (-0.27, 0.06)<br>0.216	--
Maternal Defecation (Reference: Latrine/Toilet)			
Open Area	-0.25 (-0.42, -0.08)<br>0.005	-0.04 (-0.21, 0.12)<br>0.601	--
Tuberculosis Vaccination (Reference: Vaccinated)			
Not Vaccinated	-0.15 (-0.33, 0.03)<br>0.095	0.14 (-0.04, 0.32)<br>0.121	--
Malnutrition Screening (Reference: Child Screened)			
Child Not Screened	-0.17 (-0.35, 0.00)<br>0.052	-0.08 (-0.25, 0.08)<br>0.329	--
Health-Related Knowledge (Reference: High)			
Low-to-Moderate	-0.06 (-0.24, 0.12)<br>0.498	0.05 (-0.13, 0.22)<br>0.615	--
**Immediate Level**			
Ever Breastfed (Reference: Breastfed)			
Never Breastfed	*Not assessed due to 90.5% of the sample having ever been breastfed*		
Minimum Dietary Diversity (Reference: Meets)			
Does Not Meet	-0.35 (-0.52, -0.18)<br>0.000	0.05 (-0.15, 0.25)<br>0.640	--
Fruit/Vegetable Intake the Previous Day (Reference: Consumed)			

Did Not Consume	-0.31 (-0.48, -0.14)<br>0.000	0.03 (-0.17, 0.22)<br>0.793	--
Vitamin A Food Intake the Previous Day (Reference: Consumed)			
Did Not Consume	-0.28 (-0.45, -0.11)<br>0.002	-0.04 (-0.22, 0.14)<br>0.691	--
Iron-Rich Food Intake the Previous Day (Reference: Consumed)			
Did Not Consume	-0.26 (-0.43, -0.08)<br>0.004	-0.03 (-0.21, 0.14)<br>0.706	--
Egg/Flesh Food Intake the Previous Day (Reference: Consumed)			
Did Not Consume	-0.48 (-0.65, -0.31)<br>0.000	-0.22 (-0.41, -0.03)<br>0.022	-0.24 (-0.43, -0.05)<br>0.014
Sugary Food Intake the Previous Day (Reference: Not Consumed)			
Consumed	0.21 (0.04, 0.38)<br>0.016	0.06 (-0.11, 0.23)<br>0.487	--
Illness in the Last 2 Weeks (Reference: No Illness)			
Illness	-0.09 (-0.27, 0.10)<br>0.367	-0.08 (-0.25, 0.10)<br>0.400	--
Maternal MUAC (Reference: 24-29.5 cm)			
Underweight (<24 cm)	-0.10 (-0.32, 0.13)<br>0.404	-0.09 (-0.30, 0.12)<br>0.422	--
Overweight (>29.5 cm)	0.06 (-0.14, 0.26)<br>0.550	0.10 (-0.09, 0.29)<br>0.303	--
Maternal Age (Reference: 24-34 Years)			
Younger (<24)	0.10 (-0.17, 0.37)<br>0.476	0.09 (-0.16, 0.35)<br>0.470	--
Older (35+)	0.04 (-0.14, 0.22)<br>0.659	0.09 (-0.08, 0.26)<br>0.316	--

CI – confidence interval, cm – centimetres, CU5 – children under five, M2M – mother-to-mother, MUAC – mid-upper arm circumference, NFI – non-food item

*Note that final adjusted models may have a smaller sample size due to a few variables missing some observations. Green indicates statistically significant at $p \leq 0.05$. Orange indicates marginally statistically significant from $0.05 < p \leq 0.10$.

† Adjusting for child age, sex, household region, and trial arm.

‡ Corresponding to Integrated Food Security Phase Classification 1/2, i.e. Minimal/Stressed.

§ Corresponding to Integrated Food Security Phase Classification 3/4/5, i.e. Crisis/Emergency/Famine.

Table S10. Midline Hiran Region CU5 Model: Correlates of Weight-for-Height Z-Score (n=532)*

	**Outcome: WHZ (Weight-for-Height Z-Score for children under five)**		
	**Unadjusted Bivariate Linear Regression**	**Major Confounder-Adjusted Linear Regression†**	**Final Multivariable Linear Regression (Hierarchical Approach)†**
	Coefficient (95% CI)<br>p-value	Coefficient (95% CI)<br>p-value	Coefficient (95% CI)<br>p-value
**Basic/Enabling Level**			
Wealth/Asset Index (Reference: Not Poor)			
Poor	-0.05 (-0.26, 0.16)<br>0.611	-0.06 (-0.26, 0.14)<br>0.547	--
Health and Nutrition-Related Expenditure (Reference: Middle 50%)			
Lower 25%	-0.01 (-0.29, 0.27)<br>0.947	0.06 (-0.20, 0.32)<br>0.665	--

Upper 25%	0.10 (-0.11, 0.32)<br>0.344	0.11 (-0.09, 0.31)<br>0.285	--
Decision-Making around Income, Purchases, & Healthcare (Reference: Joint)			
Maternal	-0.38 (-0.62, -0.14)<br>0.002	-0.27 (-0.50, -0.03)<br>0.024	-0.27 (-0.50, -0.03)<br>0.024
Paternal	-0.22 (-0.45, -0.00)<br>0.049	-0.17 (-0.38, 0.04)<br>0.116	-0.17 (-0.38, 0.04)<br>0.116
**Intervention Level**			
Attending M2M Groups (Reference: Attended)			
Did Not Attend	0.03 (-0.17, 0.23)<br>0.791	0.10 (-0.10, 0.30)<br>0.326	--
NFI Kits (Reference: Received)			
Not Received	*Not assessed due to 79.1% of the sample not receiving NFI kits*		
**Underlying Level**			
Household Hunger Scale (Reference: Little-to-No Hunger)‡			
Moderate-to-Severe Hunger§	-0.07 (-0.27, 0.12)<br>0.473	-0.02 (-0.21, 0.16)<br>0.820	--
Food Consumption Score (Reference: Acceptable)‡			
Unacceptable§	-0.21 (-0.41, -0.00)<br>0.045	-0.19 (-0.38, 0.01)<br>0.060	--
Reduced Consumption Scale Index (Reference: <19)‡			
≥19§	*Not assessed due to 88.1% having a rCSI <19*		
Household Crowding (Reference: Not Crowded)			
Crowded	-0.02 (-0.22, 0.19)<br>0.863	-0.05 (-0.24, 0.15)<br>0.619	--
Number of CU5 (Reference: 1 Child)			
2+ Children	0.58 (0.39, 0.78)<br>0.000	0.59 (0.40, 0.79)<br>0.000	0.27 (0.06, 0.48)<br>0.011
Water Treatment (Reference: Treats Water)			
Does Not Treat Water	0.06 (-0.15, 0.26)<br>0.575	0.04 (-0.15, 0.24)<br>0.665	--
Water Source (Reference: Piped Water)			
Not Piped	*Not assessed due to 80.9% of the sample not having piped water*		
Household Defecation (Reference: Latrine/Toilet)			
Open Area	-0.48 (-0.68, -0.28)<br>0.000	-0.40 (-0.59, -0.20)<br>0.000	-0.40 (-0.60, -0.20)<br>0.000
Child Stool Disposal (Reference: Latrine/Toilet)			
Open Area	-0.44 (-0.63, -0.25)<br>0.000	-0.35 (-0.54, -0.16)<br>0.000	--
Maternal Defecation (Reference: Latrine/Toilet)			
Open Area	-0.37 (-0.57, -0.18)<br>0.000	-0.29 (-0.49, -0.10)<br>0.003	--
Tuberculosis Vaccination (Reference: Vaccinated)			
Not Vaccinated	*Not assessed due to 75.1% of the sample not being vaccinated against tuberculosis*		
Malnutrition Screening (Reference: Child Screened)			
Child Not Screened	0.05 (-0.14, 0.25)<br>0.603	0.02 (-0.17, 0.21)<br>0.817	--
Health and Nutrition Knowledge (Reference: High)			
Low-to-Moderate	0.11 (-0.09, 0.31)<br>0.271	0.09 (-0.10, 0.28)<br>0.335	--
**Immediate Level**			
Ever Breastfed (Reference: Breastfed)			
Never Breastfed	-0.21 (-0.43, 0.01)	-0.13 (-0.34, 0.08)	

	0.067	0.222	
Minimum Dietary Diversity (Reference: Meets)			
Does Not Meet	*Not assessed due to 83.1% of the sample not meeting dietary diversity*		
Fruit/Vegetable Intake the Previous Day (Reference: Consumed)			
Did Not Consume	-0.05 (-0.26, 0.17)<br>0.679	-0.01 (-0.21, 0.20)<br>0.960	--
Vitamin A Food Intake the Previous Day (Reference: Consumed)			
Did Not Consume	-0.02 (-0.22, 0.17)<br>0.838	-0.04 (-0.22, 0.15)<br>0.703	--
Iron-Rich Food Intake the Previous Day (Reference: Consumed)			
Did Not Consume	-0.09 (-0.28, 0.11)<br>0.384	-0.15 (-0.33, 0.03)<br>0.103	--
Egg/Flesh Food Intake the Previous Day (Reference: Consumed)			
Did Not Consume	-0.13 (-0.34, 0.08)<br>0.218	-0.18 (-0.38, 0.02)<br>0.072	--
Sugary Food Intake the Previous Day (Reference: Not Consumed)			
Consumed	0.03 (-0.17, 0.22)<br>0.793	0.04 (-0.14, 0.22)<br>0.658	--
Illness in the Last 2 Weeks (Reference: No Illness)			
Illness	-0.02 (-0.23, 0.19)<br>0.863	-0.06 (-0.26, 0.14)<br>0.549	--
Maternal MUAC (Reference: 24-29.5 cm)			
Underweight (<24 cm)	-0.32 (-0.60, -0.04)<br>0.023	-0.36 (-0.62, -0.10)<br>0.006	-0.39 (-0.65, -0.13)<br>0.004
Overweight (>29.5 cm)	0.28 (0.06, 0.51)<br>0.014	0.23 (0.01, 0.44)<br>0.039	0.12 (-0.09, 0.34)<br>0.258
Maternal Age (Reference: 24-34 Years*)*			
Younger (<24)	-0.18 (-0.48, 0.12)<br>0.244	-0.20 (-0.48, 0.08)<br>0.164	--
Older (35+)	-0.06 (-0.26, 0.15)<br>0.593	0.04 (-0.16, 0.24)<br>0.679	--

CI – confidence interval, cm – centimetres, CU5 – children under five, M2M – mother-to-mother, MUAC – mid-upper arm circumference, NFI – non-food item
*Note that final adjusted models may have a smaller sample size due to a few variables missing some observations. Green indicates statistically significant at $p \leq 0.05$.
† Adjusting for child age, sex, household region, and trial arm.
‡ Corresponding to Integrated Food Security Phase Classification 1/2, i.e. Minimal/Stressed.
§ Corresponding to Integrated Food Security Phase Classification 3/4/5, i.e. Crisis/Emergency/Famine.

Table S11. Midline Bay Region CU5 Model: Correlates of Weight-for-Height Z-Score (n=401)*

	**Outcome: WHZ (Weight-for-Height Z-Score for children under five)**		
	**Unadjusted Bivariate Linear Regression**	**Major Confounder-Adjusted Linear Regression†**	**Final Multivariable Linear Regression (Hierarchical Approach)†**
	Coefficient (95% CI)<br>p-value	Coefficient (95% CI)<br>p-value	Coefficient (95% CI)<br>p-value
**Basic/Enabling Level**			
Wealth/Asset Index (Reference: Not Poor)			
Poor	*Not assessed due to 81.6% of the sample being in the poor category*		
Health and Nutrition-Related Expenditure (Reference: Middle 50%)			
Lower 25%	-0.09 (-0.31, 0.13)<br>0.436	-0.07 (-0.30, 0.16)<br>0.561	--

Upper 25%	-0.10 (-0.41, 0.21)<br>0.515	-0.10 (-0.41, 0.21)<br>0.541	--
Decision-Making around Income, Purchases, & Healthcare (Reference: joint)			
Maternal	0.17 (-0.08, 0.43)<br>0.188	0.18 (-0.08, 0.44)<br>0.180	--
Paternal	0.18 (-0.16, 0.52)<br>0.295	0.23 (-0.11, 0.57)<br>0.187	--
**Intervention Level**			
Attending M2M Groups (Reference: Attended)			
Did Not Attend	-0.00 (-0.21, 0.21)<br>0.994	0.04 (-0.18, 0.25)<br>0.747	--
NFI Kits (Reference: Received)			
Not Received	*Not assessed due to 85.5% of the sample not receiving NFI kits*		
**Underlying Level**			
Household Hunger Scale (Reference: Little-to-No Hunger)‡			
Moderate-to-Severe Hunger§	*Not assessed due to 94.0% of the sample having little-to-no hunger*		
Food Consumption Score (Reference: Acceptable)‡			
Unacceptable§	0.20 (-0.02, 0.43)<br>0.079	0.19 (-0.04, 0.41)<br>0.108	--
Reduced Consumption Score Index (Reference: <19)‡			
≥19§	-0.17 (-0.37, 0.04)<br>0.114	-0.16 (-0.36, 0.05)<br>0.132	--
Household Crowding (Reference: Not Crowded)			
Crowded	*Not assessed due to 94.2% of the sample not having a crowded household*		
Number of CU5 (Reference: 1 Child)			
2+ Children	*Not assessed due to 82.5% of the sample having only 1 child under 5 in the home*		
Water Treatment (Reference: Treats Water)			
Does Not Treat Water	*Not assessed due to 98.3% of the sample treating water with an appropriate method*		
Water Source (Reference: Piped Water)			
Not Piped	0.14 (-0.08, 0.37)<br>0.215	0.13 (-0.10, 0.36)<br>0.265	--
Household Defecation (Reference: Latrine/Toilet)			
Open Area	*Not assessed due to 90.8% of the sample not practicing open defecation*		
Child Stool Disposal (Reference: Latrine/Toilet)			
Open Area	-0.00 (-0.22, 0.21)<br>0.965	0.03 (-0.19, 0.25)<br>0.798	--
Maternal Defecation (Reference: Latrine/Toilet)			
Open Area	*Not assessed due to 83% of the mothers using a latrine/toilet*		
Tuberculosis Vaccination (Reference: Vaccinated)			
Not Vaccinated	*Not assessed due to 75.8% of the sample being vaccinated against tuberculosis*		
Malnutrition Screening (Reference: Child Screened)			
Child Not Screened	-0.01 (-0.22, 0.19)<br>0.886	-0.02 (-0.23, 0.18)<br>0.810	
Health-Related Knowledge (Reference: High)			
Low-to-Moderate	*Not assessed due to 78.6% of the sample having high health-related knowledge*		
**Immediate Level**			
Ever Breastfed (Reference: Breastfed)			
Never Breastfed	*Not assessed due to 91.3% of the sample having ever been breastfed*		
Minimum Dietary Diversity (Reference: Meets)			
Does Not Meet	0.02 (-0.19, 0.24)	0.02 (-0.20, 0.24)	--

	0.841	0.867	
Fruit/Vegetable Intake the Previous Day (Reference: Consumed)			
Did Not Consume	*Not assessed due to 86.8% consuming fruit/vegetables the previous day*		
Vitamin A Food Intake the Previous Day (Reference: Consumed)			
Did Not Consume	*Not assessed due to 87.8% consuming Vitamin A foods the previous day*		
Iron-Rich Food Intake the Previous Day (Reference: Consumed)			
Did Not Consume	*Not assessed due to 78.6% of the sample consuming Iron-rich foods the previous day*		
Egg/Flesh Food Intake the Previous Day (Reference: Consumed)			
Did Not Consume	0.09 (-0.12, 0.29)<br>0.390	0.09 (-0.11, 0.30)<br>0.382	--
Sugary Food Intake the Previous Day (Reference: Not Consumed)			
Consumed	-0.09 (-0.30, 0.13)<br>0.444	-0.06 (-0.28, 0.16)<br>0.596	--
Illness in the Last 2 Weeks (Reference: No Illness)			
Illness	-0.27 (-0.47, -0.06)<br>0.013	-0.26 (-0.47, -0.06)<br>0.013	-0.26 (-0.47, -0.06)<br>0.013
Maternal MUAC (Reference: 24-29.5 cm)			
Underweight (<24 cm)	-0.08 (-0.37, 0.22)<br>0.610	-0.06 (-0.35, 0.24)<br>0.694	--
Overweight (>29.5 cm)	0.03 (-0.20, 0.26)<br>0.822	0.02 (-0.21, 0.25)<br>0.853	--
Maternal Age (Reference: 24-34)			
Younger (<24)	0.07 (-0.21, 0.36)<br>0.609	0.08 (-0.21, 0.37)<br>0.582	--
Older (35+)	0.06 (-0.16, 0.28)<br>0.571	0.07 (-0.15, 0.29)<br>0.532	--

CI – confidence interval, cm – centimetres, CU5 – children under five, M2M – mother-to-mother, MUAC – mid-upper arm circumference, NFI – non-food item

*Note that final adjusted models may have a smaller sample size due to a few variables missing some observations. Green indicates statistically significant at $p \leq 0.05$.

† Adjusting for child age, sex, household region, and trial arm.

‡ Corresponding to Integrated Food Security Phase Classification 1/2, i.e. Minimal/Stressed.

§ Corresponding to Integrated Food Security Phase Classification 3/4/5, i.e. Crisis/Emergency/Famine.

Table S12. Endline Hiran Region CU5 Model: Correlates of Weight-for-Height Z-Score (n=468)*

	**Outcome: WHZ (Weight-for-Height Z-Score for children under five)**		
	**Unadjusted Bivariate Linear Regression**	**Major Confounder-Adjusted Linear Regression†**	**Final Multivariable Linear Regression (Hierarchical Approach)†**
	Coefficient (95% CI)<br>p-value	Coefficient (95% CI)<br>p-value	Coefficient (95% CI)<br>p-value
**Basic/Enabling Level**			
Wealth/Asset Index (Reference: Not Poor)			
Poor	*Not assessed due to 90.8% of the sample being in the "not poor" category*		
Health and Nutrition-Related Expenditure (Reference: Middle 50%)			
Lower 25%	-0.16 (-0.43, 0.11)<br>0.248	-0.16 (-0.41, 0.10)<br>0.224	--
Upper 25%	0.07 (-0.16, 0.30)<br>0.552	0.15 (-0.07, 0.38)<br>0.180	--

Head of Household (Reference: Mother)			
Father or Other	0.13 (-0.08, 0.35)<br>0.224	0.12 (-0.09, 0.33)<br>0.257	--
Decision-Making around Income, Purchases, & Healthcare (Reference: Joint)			
Maternal	-0.07 (-0.31, 0.17)<br>0.561	-0.06 (-0.28, 0.17)<br>0.621	--
Paternal	0.04 (-0.23, 0.31)<br>0.778	0.01 (-0.25, 0.27)<br>0.945	--
Maternal Education (Reference: Some Education)			
No Education	-0.13 (-0.33, 0.08)<br>0.240	-0.10 (-0.30, 0.10)<br>0.312	--
Displacement in the Last 3 Months (Reference: Not Displaced)			
Displaced	*Not assessed due to 98.7% of the sample not being displaced in the last 3 months*		
**Intervention Level**			
Attending M2M Groups (Reference: Attended)			
Did Not Attend	0.05 (-0.18, 0.28)<br>0.666	0.19 (-0.04, 0.41)<br>0.105	--
NFI Kits (Reference: Received)			
Not Received	0.16 (-0.04, 0.36)<br>0.120	0.21 (0.01, 0.40)<br>0.035	0.21 (0.01, 0.40)<br>0.035
**Underlying Level**			
Household Hunger Scale (Reference: Little-to-No Hunger)‡			
Moderate-to-Severe Hunger§	-0.04 (-0.25, 0.17)<br>0.683	-0.01 (-0.21, 0.19)<br>0.943	--
Food Consumption Score (Reference: Acceptable)‡			
Unacceptable§	-0.07 (-0.27, 0.14)<br>0.516	-0.04 (-0.23, 0.16)<br>0.720	--
Reduced Coping Strategy Index (Reference: <19)‡			
≥19§	-0.16 (-0.38, 0.05)<br>0.134	-0.06 (-0.27, 0.14)<br>0.534	--
Number of CU5 (Reference: 1 Child)			
2+ Children	0.36 (0.15, 0.58)<br>0.001	0.08 (-0.15, 0.30)<br>0.493	--
Water Treatment (Reference: Treats Water)			
Does Not Treat Water	*Not assessed due to 84.8% of the sample treating water*		
Water Source (Reference: Piped Water)			
Not Piped	-0.07 (-0.29, 0.15)<br>0.536	-0.08 (-0.30, 0.13)<br>0.454	--
Household Defecation (Reference: Latrine/Toilet)			
Open Area	-0.15 (-0.38, 0.08)<br>0.190	-0.03 (-0.26, 0.19)<br>0.783	--
Child Stool Disposal (Reference: Latrine/Toilet)			
Open Area	-0.15 (-0.36, 0.05)<br>0.143	-0.09 (-0.29, 0.11)<br>0.385	--
Maternal Defecation (Reference: Latrine/Toilet)			
Open Area	-0.28 (-0.48, -0.08)<br>0.007	-0.19 (-0.38, 0.00)<br>0.055	-0.17 (-0.36, 0.01)<br>0.065
Tuberculosis Vaccination (Reference: Vaccinated)			
Not Vaccinated	0.04 (-0.16, 0.24)<br>0.714	0.13 (-0.07, 0.32)<br>0.196	--
Malnutrition Screening (Reference: Child Screened)			
Child Not Screened	-0.12 (-0.34, 0.09)<br>0.253	-0.08 (-0.28, 0.13)<br>0.453	
Health-Related Knowledge (Reference: High)			

Low-to-Moderate	-0.06 (-0.26, 0.15)<br>0.596	-0.02 (-0.22, 0.18)<br>0.871	--
**Immediate Level**			
Ever Breastfed (Reference: Breastfed)			
Never Breastfed	*Not assessed due to 90.6% of the sample ever being breastfed*		
Minimum Dietary Diversity (Reference: Meets)			
Does Not Meet	*Not assessed due to 83.8% of the sample not being minimum dietary diversity*		
Fruit/Vegetable Intake the Previous Day (Reference: Consumed)			
Did Not Consume	0.23 (0.01, 0.46)<br>0.039	0.12 (-0.10, 0.34)<br>0.282	--
Vitamin A Food Intake the Previous Day (Reference: Consumed)			
Did Not Consume	0.04 (-0.16, 0.25)<br>0.694	-0.01 (-0.21, 0.18)<br>0.900	--
Iron-Rich Food Intake the Previous Day (Reference: Consumed)			
Did Not Consume	0.14 (-0.07, 0.34)<br>0.184	0.12 (-0.07, 0.31)<br>0.224	--
Egg/Flesh Food Intake the Previous Day (Reference: Consumed)			
Did Not Consume	*Not assessed due to 78.6% of the sample not eating egg and/or flesh foods the previous day*		
Sugary Food Intake the Previous Day (Reference: Not Consumed)			
Consumed	-0.04 (-0.24, 0.16)<br>0.712	0.01 (-0.18, 0.20)<br>0.919	--
Illness in the Last 2 Weeks (Reference: No Illness)			
Illness	-0.04 (-0.26, 0.18)<br>0.702	-0.04 (-0.25, 0.17)<br>0.685	--
Maternal MUAC (Reference: 24-29.5 cm)			
Underweight (<24 cm)	-0.12 (-0.38, 0.15)<br>0.383	-0.10 (-0.35, 0.15)<br>0.451	--
Overweight (>29.5 cm)	0.17 (-0.07, 0.40)<br>0.171	0.19 (-0.04, 0.42)<br>0.111	--
Maternal Age (Reference: 24-34 Years)			
Younger (<24)	0.01 (-0.31, 0.33)<br>0.934	-0.01 (-0.32, 0.29)<br>0.935	--
Older (35+)	-0.07 (-0.29, 0.14)<br>0.510	0.03 (-0.18, 0.24)<br>0.792	--

CI – confidence interval, cm – centimetres, CU5 – children under five, M2M – mother-to-mother, MUAC – mid-upper arm circumference, NFI – non-food item

*Note that final adjusted models may have a smaller sample size due to a few variables missing some observations. Green indicates statistically significant at $p \leq 0.05$. Orange indicates marginally statistically significant from $0.05 < p \leq 0.10$.

† Adjusting for child age, sex, household region, and trial arm.

‡ Corresponding to Integrated Food Security Phase Classification 1/2, i.e. Minimal/Stressed.

§ Corresponding to Integrated Food Security Phase Classification 3/4/5, i.e. Crisis/Emergency/Famine.

Table S13. Endline Bay CU5 Model: Correlates of Weight-for-Height Z-Score (n=364)*

	**Outcome: WHZ (Weight-for-Height Z-Score for children under five)**		
	**Unadjusted Bivariate Linear Regression**	**Major Confounder-Adjusted Linear Regression†**	**Final Multivariable Linear Regression (Hierarchical Approach)†**
	Coefficient (95% CI)<br>p-value	Coefficient (95% CI)<br>p-value	Coefficient (95% CI)<br>p-value
**Basic/Enabling Level**			

Wealth/Asset Index (Reference: Not Poor)			
Poor	*Not assessed due to 79.7% of the sample being in the "poor" category*		
Health and Nutrition-Related Expenditure (Reference: Middle 50%)			
Lower 25%	-0.08 (-0.33, 0.16)<br>0.517	-0.06 (-0.30, 0.19)<br>0.640	--
Upper 25%	0.18 (-0.12, 0.48)<br>0.247	0.16 (-0.14, 0.46)<br>0.290	--
Head of Household (Reference: Mother)			
Father or Other	-0.19 (-0.41, 0.02)<br>0.080	-0.20 (-0.43, 0.02)<br>0.070	-0.31 (-0.56, -0.06)<br>0.014
Decision-Making around Income, Purchases, & Healthcare (Reference: Joint)			
Maternal	-0.27 (-0.56, 0.02)<br>0.069	-0.24 (-0.53, 0.05)<br>0.110	-0.41 (-0.74, -0.09)<br>0.012
Paternal	-0.28 (-0.68, 0.12)<br>0.172	-0.35 (-0.76, 0.06)<br>0.092	-0.26 (-0.67, 0.16)<br>0.222
Maternal Education (Reference: Some Education)			
No Education	*Not assessed due to 76.1% of the sample's mothers not having any education*		
Displacement in the Last 3 Months (Reference: Not Displaced)			
Displaced	0.05 (-0.18, 0.29)<br>0.666	0.01 (-0.24, 0.26)<br>0.926	--
**Intervention Level**			
Attending M2M Groups (Reference: Attended)			
Did Not Attend	0.18 (-0.05, 0.42)<br>0.129	0.15 (-0.10, 0.40)<br>0.252	--
NFI Kits (Reference: Received)			
Not Received	*Not assessed due to 78.6% of the sample not receiving NFI kits*		
**Underlying Level**			
Household Hunger Scale (Reference: Little-to-No Hunger)‡			
Moderate-to-Severe Hunger§	0.13 (-0.11, 0.36)<br>0.286	0.15 (-0.09, 0.38)<br>0.217	--
Food Consumption Score (Reference: Acceptable)‡			
Unacceptable§	-0.17 (-0.39, 0.05)<br>0.131	-0.14 (-0.37, 0.09)<br>0.221	--
Reduced Coping Strategy Index (Reference: <19)‡			
≥19§	-0.15 (-0.39, 0.10)<br>0.245	-0.11 (-0.36, 0.14)<br>0.380	
Number of CU5 (Reference: 1 Child)			
2+ Children	*Not assessed due to 84.7% of the sample having 1 child-under-5 in the household*		
Water Treatment (Reference: Treats Water)			
Does Not Treat Water	*Not assessed due to 92.3% of the sample treating water*		
Water Source (Reference: Piped Water)			
Not Piped	0.07 (-0.15, 0.28)<br>0.555	0.05 (-0.18, 0.28)<br>0.686	--
Household Defecation (Reference: Latrine/Toilet)			
Open Area	*Not assessed due to 78.6% of the sample not practicing open defecation*		
Child Stool Disposal (Reference: Latrine/Toilet)			
Open Area	-0.11 (-0.32, 0.11)<br>0.336	-0.08 (-0.31, 0.14)<br>0.473	--
Maternal Defecation (Reference: Latrine/Toilet)			
Open Area	0.04 (-0.20, 0.28)<br>0.728	0.05 (-0.19, 0.29)<br>0.698	--
Tuberculosis Vaccination (Reference: Vaccinated)			
Not Vaccinated	*Not assessed due to 83.3% of the sample being vaccinated against tuberculosis*		
Malnutrition Screening (Reference: Child Screened)			

Child Not Screened	-0.08 (-0.30, 0.14)<br>0.456	-0.09 (-0.31, 0.13)<br>0.404	
Health-Related Knowledge (Reference: High)			
Low-to-Moderate	0.21 (-0.03, 0.45)<br>0.089	0.18 (-0.08, 0.44)<br>0.164	--
**Immediate Level**			
Ever Breastfed (Reference: Breastfed)			
Never Breastfed	*Not assessed due to 88.2% of the sample having ever been breastfed*		
Minimum Dietary Diversity (Reference: Meets)			
Does Not Meet	-0.08 (-0.33, 0.16)<br>0.506	-0.09 (-0.34, 0.16)<br>0.480	--
Fruit/Vegetable Intake the Previous Day (Reference: Consumed)			
Did Not Consume	*Not assessed due to 84.7% of the sampling eating fruits/vegetables the previous day*		
Vitamin A Food Intake the Previous Day (Reference: Consumed)			
Did Not Consume	*Not assessed due to 82.2% of the sample consuming Vitamin A-rich foods the previous day*		
Iron-Rich Food Intake the Previous Day (Reference: Consumed)			
Did Not Consume	*Not assessed due to 79.2% of the sample consuming Iron-rich foods the previous day*		
Eggs/Flesh Food Intake the Previous Day (Reference: Consumed)			
Did Not Consume	-0.10 (-0.34, 0.14)<br>0.429	-0.10 (-0.35, 0.15)<br>0.443	--
Sugar Food Intake the Previous Day (Reference: Not Consumed)			
Consumed	0.05 (-0.19, 0.28)<br>0.698	0.02 (-0.22, 0.26)<br>0.845	--
Illness in the Last 2 Weeks (Reference: No Illness)			
Illness	-0.23 (-0.46, 0.01)<br>0.060	-0.22 (-0.46, 0.01)<br>0.063	--
Maternal MUAC (Reference: 24-29.5 cm)			
Underweight (<24 cm)	0.14 (-0.14, 0.42)<br>0.324	0.12 (-0.16, 0.40)<br>0.396	0.12 (-0.16, 0.39)<br>0.409
Overweight (>29.5 cm)	0.25 (-0.01, 0.50)<br>0.059	0.24 (-0.02, 0.49)<br>0.069	0.28 (0.03, 0.54)<br>0.030
Maternal Age (Reference: 24-34 Years)			
Younger (<24)	0.04 (-0.26, 0.35)<br>0.775	0.04 (-0.27, 0.35)<br>0.797	--
Older (35+)	0.06 (-0.18, 0.30)<br>0.612	0.08 (-0.16, 0.32)<br>0.526	--

CI – confidence interval, cm – centimetres, CU5 – children under five, M2M – mother-to-mother, MUAC – mid-upper arm circumference, NFI – non-food item

*Note that final adjusted models may have a smaller sample size due to a few variables missing some observations. Green indicates statistically significant at $p \leq 0.05$.

† Adjusting for child age, sex, household region, and trial arm.

‡ Corresponding to Integrated Food Security Phase Classification 1/2, i.e. Minimal/Stressed.

§ Corresponding to Integrated Food Security Phase Classification 3/4/5, i.e. Crisis/Emergency/Famine.

Correlates of Wasting (MUAC<23cm)

Basic/Enabling
- Decision-Making[M]

Intervention
- M2M Groups[E]

Underlying
- Household Hunger Scale[M]
- Food Consumption Score[M]
- Child Stool Disposal[M]
- Open Defecation[E]

Immediate
- Age[M]

Figure S6. Correlates of Mothers' Wasting

"M" superscript indicates the variable was a statistically significant correlate in the given model at midline, and "E" superscript indicates the variable was a correlate in the given model at endline. Note that this image depicts statistically significant correlates from the final adjusted models at the α = 0.05 level; additional correlates may have been marginally statistically significant or statistically significant in the unadjusted or basic confounder-adjusted models.

Table S14. Midline Overall Maternal MUAC Model: Correlates of Wasting (n=1066)*

	**Outcome: Wasting (Mid-upper arm circumference <23 cm of mothers)**		
	**Unadjusted Bivariate Poisson Regression**	**Major Confounder-Adjusted Poisson Regression†**	**Final Multivariable Poisson Regression (Hierarchical Approach)†**
	Relative Risk (95% CI) p-value	Relative Risk (95% CI) p-value	Relative Risk (95% CI) p-value
**Basic/Enabling Level**			
Wealth/Asset Index (Reference: Not Poor)			
Poor	1.16 (0.76, 1.77)<br>0.486	1.42 (0.89, 2.25)<br>0.142	--
Health- and Nutrition-Related Expenditure (Reference: Middle 50%)			
Lower 25%	1.42 (0.86, 2.34)<br>0.172	1.49 (0.88, 2.51)<br>0.136	1.59 (0.94, 2.68)<br>0.085
Upper 25%	1.30 (0.78, 2.18)<br>0.310	1.23 (0.73, 2.06)<br>0.441	1.20 (0.71, 2.02)<br>0.495
Decision-Making around Income, Purchases, & Healthcare (Reference: Joint)			
Maternal	0.54 (0.29, 1.04)<br>0.066	0.52 (0.27, 0.99)<br>0.047	0.49 (0.26, 0.95)<br>0.034
Paternal	1.00 (0.61, 1.66)<br>0.988	0.90 (0.53, 1.52)<br>0.689	0.88 (0.52, 1.48)<br>0.631
Region (Reference: Bay)			
Hiran	1.33 (0.86, 2.06)<br>0.203	1.35 (0.86, 2.11)<br>0.187	1.48 (0.92, 2.40)<br>0.108
**Intervention Level**			

Attending M2M Groups (Reference: Attended)			
Did Not Attend	1.27 (0.83, 1.94)<br>0.274	1.25 (0.78, 1.99)<br>0.354	--
NFI Kits (Reference: Received)			
Not Received	*Not assessed due to 81.9% of the sample not receiving NFI kits*		
**Underlying Level**			
Household Hunger Scale (Reference: Little-to-No Hunger)‡			
Moderate-to-Severe Hunger§	1.84 (1.19, 2.82)<br>0.006	1.84 (1.13, 2.98)<br>0.014	1.79 (1.09, 2.95)<br>0.021
Food Consumption Score (Reference: Acceptable)‡			
Unacceptable§	1.68 (1.10, 2.56)<br>0.017	1.63 (1.07, 2.50)<br>0.024	1.60 (1.04, 2.47)<br>0.032
Reduced Coping Strategy Index (Reference: <19)‡			
≥19§	*Not assessed due to 77.5% of the sample having a rCSI <19*		
Household Crowding (Reference: Not Crowded)			
Crowded	1.21 (0.79, 1.86)<br>0.382	1.08 (0.63, 1.83)<br>0.784	--
Number of CU5 (Reference: 1 Child)			
2+ Children	0.82 (0.49, 1.36)<br>0.432	0.78 (0.46, 1.30)<br>0.341	--
Water Treatment (Reference: Treats Water)			
Does Not Treat Water	*Not assessed due to 78.5% of the sample treating water with an appropriate method*		
Water Source (Reference: Piped Water)			
Not Piped	*Not assessed due to 79.7% of the sample not having household piped water*		
Household Defecation (Reference: Latrine/Toilet)			
Open Area	*Not assessed due to 76.2% of the sample not practicing open defecation*		
Child Stool Disposal (Reference: Latrine/Toilet)			
Open Area	2.15 (1.28, 3.61)<br>0.004	2.23 (1.32, 3.77)<br>0.003	2.04 (1.20, 3.49)<br>0.009
Maternal Defecation (Reference: Latrine/Toilet)			
Open Area	2.23 (1.46, 3.39)<br>0.000	2.26 (1.45, 3.52)<br>0.000	--
Malnutrition Screening (Reference: Mother Screened)			
Mother Not Screened	0.70 (0.44, 1.10)<br>0.124	0.74 (0.46, 1.18)<br>0.206	--
Health-Related Knowledge (Reference: High)			
Low-to-Moderate	1.48 (0.96, 2.26)<br>0.073	1.44 (0.93, 2.22)<br>0.102	--
**Immediate Level**			
Maternal Age (Reference: 24-34 Years)			
Younger (<24)	1.19 (0.71, 1.99)<br>0.520	1.21 (0.72, 2.04)<br>0.469	1.26 (0.75, 2.14)<br>0.382
Older (35+)	0.40 (0.23, 0.69)<br>0.001	0.39 (0.23, 0.68)<br>0.001	0.40 (0.23, 0.70)<br>0.001

CI – confidence interval, cm – centimetres, CU5 – children under five, M2M – mother-to-mother, NFI – non-food item
*Note that final adjusted models may have a smaller sample size due to a few variables missing some observations. Green indicates statistically significant at $p \leq 0.05$. Orange indicates marginally statistically significant from $0.05 < p \leq 0.10$. Pearson goodness-of-fit p-value for final fully adjusted model = 0.6294.
† Adjusting for household region and trial arm.
‡ Corresponding to Integrated Food Security Phase Classification 1/2, i.e. Minimal/Stressed.
§ Corresponding to Integrated Food Security Phase Classification 3/4/5, i.e. Crisis/Emergency/Famine.

Table S15. Endline Overall Maternal MUAC Model: Correlates of Wasting (n=1023)*

	**Outcome: Wasting (Mid-upper arm circumference <23 cm of mothers)**		
	**Unadjusted Bivariate Poisson Regression**	**Major Confounder-Adjusted Poisson Regression†**	**Final Multivariable Poisson Regression (Hierarchical Approach)†**
	Relative Risk (95% CI) p-value	Relative Risk (95% CI) p-value	Relative Risk (95% CI) p-value
**Basic/Enabling Level**			
Wealth/Asset Index (Reference: Not Poor)			
Poor	1.06 (0.74, 1.52) 0.747	1.32 (0.80, 2.17) 0.280	
Health- and Nutrition-Related Expenditure (Reference: Middle 50%)			
Lower 25%	1.10 (0.72, 1.69) 0.647	1.14 (0.74, 1.75) 0.546	--
Upper 25%	0.97 (0.62, 1.51) 0.881	0.93 (0.59, 1.46) 0.747	--
Head of Household (Reference: Mother)			
Father or Other	0.99 (0.69, 1.41) 0.948	1.01 (0.70, 1.45) 0.975	--
Decision-Making around Income, Purchases, & Healthcare (Reference: Joint)			
Maternal	0.75 (0.47, 1.21) 0.245	0.73 (0.45, 1.18) 0.204	--
Paternal	0.73 (0.41, 1.30) 0.281	0.69 (0.38, 1.25) 0.223	--
Maternal Education (Reference: Some Education)			
No Education	1.25 (0.83, 1.87) 0.283	1.25 (0.83, 1.87) 0.289	--
Displacement in the Last 3 Months (Reference: Not Displaced)			
Displaced	0.66 (0.36, 1.20) 0.173	0.64 (0.34, 1.21) 0.169	--
Region (Reference: Bay)			
Hiran	1.10 (0.77, 1.57) 0.603	1.10 (0.77, 1.58) 0.601	1.10 (0.77, 1.58) 0.601
**Intervention Level**			
Attending M2M Groups (Reference: Attended)			
Did Not Attend	0.60 (0.38, 0.94) 0.027	0.55 (0.35, 0.87) 0.012	0.55 (0.35, 0.87) 0.012

NFI Kits (Reference: Received)			
Not Received	1.09 (0.75, 1.57)<br>0.664	1.13 (0.77, 1.67)<br>0.538	--
**Underlying Level**			
Household Hunger Scale (Reference: Little-to-No Hunger)‡			
Moderate-to-Severe Hunger§	0.97 (0.68, 1.38)<br>0.855	0.94 (0.65, 1.38)<br>0.764	--
Food Consumption Score (Reference: Acceptable)‡			
Unacceptable§	1.20 (0.84, 1.72)<br>0.307	1.18 (0.82, 1.70)<br>0.378	--
Reduced Coping Strategy Index (Reference: <19)‡			
≥19§	1.02 (0.69, 1.51)<br>0.908	1.02 (0.69, 1.50)<br>0.930	--
Number of CU5 (Reference: 1 Child)			
2+ Children	1.00 (0.67, 1.51)<br>0.981	0.97 (0.64, 1.47)<br>0.892	--
Water Treatment (Reference: Treats Water)			
Does Not Treat Water	1.28 (0.80, 2.05)<br>0.304	1.30 (0.80, 2.09)<br>0.287	--
Water Source (Reference: Piped Water)			
Not Piped	1.12 (0.76, 1.63)<br>0.570	1.11 (0.75, 1.64)<br>0.604	--
Household Defecation (Reference: Latrine/Toilet)			
Open Area	1.37 (0.94, 2.01)<br>0.103	1.36 (0.93, 2.00)<br>0.116	1.57 (1.06, 2.33)<br>0.024
Child Stool Disposal (Reference: Latrine/Toilet)			
Open Area	0.95 (0.66, 1.35)<br>0.758	0.95 (0.66, 1.37)<br>0.790	--
Maternal Defecation (Reference: Latrine/Toilet)			
Open Area	1.14 (0.80, 1.62)<br>0.482	1.10 (0.76, 1.59)<br>0.616	--
Malnutrition Screening (Reference: Mother Screened)			
Mother Not Screened	0.88 (0.60, 1.30)<br>0.531	0.98 (0.63, 1.51)<br>0.918	--
Health-Related Knowledge (Reference: High)			
Low-to-Moderate	0.92 (0.63, 1.33)<br>0.657	0.85 (0.58, 1.25)<br>0.409	--
**Immediate Level**			
Maternal Age (Reference: 24-34 Years)			
Younger (<24)	1.23 (0.77, 1.96)<br>0.382	1.23 (0.77, 1.97)<br>0.380	--
Older (35+)	0.71 (0.47, 1.07)<br>0.104	0.72 (0.48, 1.09)<br>0.120	--

CI – confidence interval, cm – centimetres, CU5 – children under five, M2M – mother-to-mother, NFI – non-food item

*Note that final adjusted models may have a smaller sample size due to a few variables missing some observations. Green indicates statistically significant at $p \leq 0.05$. Pearson goodness-of-fit p-value for final fully adjusted model = 0.9975.

† Adjusting for household region and trial arm.

‡ Corresponding to Integrated Food Security Phase Classification 1/2, i.e. Minimal/Stressed.

§ Corresponding to Integrated Food Security Phase Classification 3/4/5, i.e. Crisis/Emergency/Famine.

References

1 Nutrition Landscape Information System, World Health Organization. Malnutrition in children. Available: https://www.who.int/data/nutrition/nlis/info/malnutrition-in-children#:~:text=These%20indicators%20are%20defined%20as%20follows%3A%201%20stunting,%28SD%29%20of%20the%20WHO%20Child%20growth%20standards%20median%3B. Accessed: 22 May 2025.

2 World Health Organization. Malnutrition. Available: https://www.who.int/health-topics/malnutrition. Accessed: 23 September 2024.

3 World Health Organization. Child malnutrition: Wasting among children under 5 years of age. Available: https://www.who.int/data/gho/indicator-metadata-registry/imr-details/302. Accessed: 23 September 2024.

4 World Health Organization, United Nations Children's Fund, World Food Programme. Global nutrition targets 2025: Wasting Policy Brief (WHO/NMH/NHD/14.8). Geneva, Switzerland: The World Health Organization; 2014. Available: https://www.who.int/publications/i/item/WHO-NMH-NHD-14.8. Accessed: 23 September 2024.

5 World Health Organization. Child growth standards. Available: https://www.who.int/tools/child-growth standards. Accessed: 23 September 2024.

6 WHO Multicentre Growth Reference Study Group. WHO Child Growth Standards based on length/height, weight and age. Acta Paediatr Suppl. 2006;450:76–85. Medline:16817681

7 Food and Nutrition Technical Assistance. Determining a Global Mid-Upper Arm Circumference Cutoff to Assess Malnutrition in Pregnant Women. Washington, DC, USA: Food and Nutrition Technical Assistance; 2016. Available: https://www.fantaproject.org/sites/default/files/resources/FANTA-MUAC-cutoffs pregnant-women-June2016.pdf.

8 Kumar P, Sinha R, Patil N, Kumar V. Relationship between mid-upper arm circumference and BMI for identifying maternal wasting and severe wasting: a cross-sectional assessment. Public Health Nutr. 2019;22(14):2548–52. Medline:31084660 doi:10.1017/S1368980019000727

9 Kumar P, Sareen N, Agrawal S, Kathuria N, Yadav S, Sethi V. Screening maternal acute malnutrition using adult mid-upper arm circumference in resource-poor settings. Indian Journal of Community Medicine. 2018;43(2):132-34. Medline:29899619 doi:10.4103/ijcm.IJCM_248_17

10 Walton S, Alier KK, Garretson S, Grounds S, Khattak Q, Tripaldi M, et al. A Cluster-Randomized Trial to Compare Effectiveness and Cost-effectiveness of Cash Plus Interventions in Preventing Child Wasting in Somalia: An Evidence-Based Methodology. J Glob Health. Forthcoming 2025. doi:10.1101/2025.02.21.25322679

11 Akseer N, Alier KK, Grounds S, Garretson S, Khattak Q, Tripaldi M, et al. The Effectiveness and Cost-Effectiveness of CashPlus Programs to Prevent Acute Malnutrition in Somalia: Evidence from a Cluster Randomized Control Trial. J Glob Health. Forthcoming 2025.

12 Directorate of National Statistics, Federal Government of Somalia. The Somali Health and Demographic Survey 2020. Mogadishu, Somalia: Directorate of National Statistics, Federal Government of Somalia; 2020. Available: https://reliefweb.int/report/somalia/somali-health-and-demographic-survey-2020. Accessed: 23 September 2024.

13 World Health Organization 2024 data.who.int. Somalia [Country overview]. 2024. Available: https://data.who.int/countries/706. Accessed: 24 September 2024.

14 Food and Agriculture Organization of the United Nations. Somalia Water and Land Information Management. Climate. Available: https://www.faoswalim.org/water/climate-somalia. Accessed: 23 September 2024.

15 United States Agency for International Development. Somalia - Complex Emergency Fact Sheet #1, Fiscal Year (FY) 2025 (December 20, 2024). Washington, D.C., USA: United States Agency for International Development; 2024. Available: https://reliefweb.int/report/somalia/somalia-complex-emergency-fact-sheet-1-fiscal-year-fy-2025-december-20-2024.

16 European Asylum Support Office. EASO Country of Origin Information Report: Somalia Security Situation. European Asylum Support Office; 2017. Available: https://reliefweb.int/report/somalia/easo-country-origin-information-report-somalia-security-situation. Accessed: 23 September 2024.

17 European Union Agency for Asylum. Bay. 2022. Available: https://euaa.europa.eu/country-guidance-somalia-2022/bay. Accessed: 23 September 2024.

18 European Union Agency for Asylum. Hiraan. 2022. Available: https://euaa.europa.eu/country-guidance-somalia-2022/hiraan. Accessed: 23 September 2024.

19 European Union Agency for Asylum. Country Guidance: Somalia. Luxembourg: European Union Agency for Asylum; 2023. Available: https://euaa.europa.eu/publications/country-guidance-somalia-august-2023.

20 Food Security and Nutrition Analysis Unit – Somalia. Somalia IPC Population Estimates: Current (Jan-March 2025). Nairobi, Kenya: Food Security and Nutrition Analysis Unit – Somalia; 2025. Available: https://reliefweb.int/report/somalia/somalia-ipc-population-estimates-current-jan-mar-2025. Accessed: 19 May 2025.

21 United Nations Office for the Coordination of Humanitarian Affairs. Somalia: 2023 Deyr Season Floods Situation Report No. 5. Mogadishu, Somalia: United Nations Office for the Coordination of Humanitarian Affairs; 2023. Available:

https://reliefweb.int/report/somalia/somalia-2023-deyr-season-floods-situation-report-no-5-24-december-2023. Accessed: 23 September 2024.

22 ACAPS. Somalia - Flooding in Baidoa (26 October 2023). Geneva, Switzerland: ACAPS; 2023. Available: https://www.acaps.org/en/countries/archives/detail/somalia-flooding-in-baidoa. Accessed: 23 September 2024.

23 ACAPS. Somalia: Key crises to watch in 2023. Geneva, Switzerland: ACAPS; 2023. Available: https://www.acaps.org/en/countries/archives/detail/somalia-key-crises-to-watch-in-2023. Accessed: 23 September 2024.

24 United Nations Refugee Agency Operational Data Portal, UNHCR-led Protection and Solutions Monitoring Network (PSMN) Somalia. Somalia: Internal Displacement – Displacements Monitored by UNHCR Protection and Solutions Monitoring Network (PSMN). Available: https://data.unhcr.org/en/dataviz/1?sv=1&geo=192. Accessed: 24 September 2024.

25 von Elm E, Altman DG, Egger M, Pocock SJ, Gotzsche PC, Vandenbroucke JP, et al. The Strengthening the Reporting of Observational Studies in Epidemiology (STROBE) Statement: guidelines for reporting observational studies. PLoS Med. 2007;4(10):e296. Medline:17941714 doi:10.1371/journal.pmed.0040296

26 People In Need, IndiKit. Ever Breastfed. Available: https://www.indikit.net/indicator/1-nutrition/4432-ever-breastfed. Accessed: 24 September 2024.

27 Data4Diets: Building Blocks for Diet-related Food Security Analysis, Version 2.0. Minimum Dietary Diversity (MDD-IYCF) for children 6-23 months old. Tufts University, Boston, Massachusetts, USA. 2023. Available: https://inddex.nutrition.tufts.edu/data4diets/indicator/minimum-dietary-diversity-mdd-iycf. Accessed: 23 September 2024.

28 Kennedy G, Ballard T, Dop M. Guidelines for measuring household and individual dietary diversity. Rome, Italy: Food and Agriculture Organization of the United Nations; 2011. Available: https://www.fao.org/fileadmin/user_upload/wa_workshop/docs/FAO-guidelines-dietary-diversity2011.pdf. Accessed: 23 September 2024.

29 World Health Organization, United Nations Children’s Fund. Indicators for assessing infant and young child feeding practices: Definitions and measurement methods. Geneva, Switzerland: World Health Organization, United Nations Children’s Fund; 2021. Available: https://www.who.int/publications/i/item/9789240018389. Accessed: 23 September 2024.

30 People In Need, IndiKit. Egg and/or Flesh Food Consumption by Children. Available: https://www.indikit.net/indicator/1-nutrition/4451-egg-and-or-flesh-food-consumption-by-children. Accessed: 24 September 2024.

31 People In Need, IndiKit. Zero Vegetable/Fruit Consumption by Children. Available: https://www.indikit.net/indicator/1-nutrition/4454-zero-vegetable-fruit-consumption-by-children. Accessed: 24 September 2024.

32 People In Need, IndiKit. Consumption of Vitamin A Rich Foods Among Children. Available: https://www.indikit.net/indicator/1-nutrition/272-consumption-of-vitamin-a-rich-foods-among-children. Accessed: 24 September 2024.

33 People In Need, IndiKit. Consumption of Iron-Rich Foods Among Children. Available: https://www.indikit.net/indicator/1-nutrition/274-consumption-of-iron-rich-foods-among-children. Accessed: 24 September 2024.

34 People In Need, IndiKit. Unhealthy Food Consumption by Children. Available: https://www.indikit.net/indicator/1-nutrition/4453-unhealthy-food-consumption-by-children. Accessed: 24 September 2024.

35 The Demographic and Health Surveys Program, ICF, United States Agency for International Development. Wealth Index Construction. Available: https://dhsprogram.com/topics/wealth-index/Wealth-Index-Construction.cfm. Accessed: 24 September 2024.

36 Cooperative for Assistance and Relief Everywhere. The Coping Strategies Index. Atlanta, Georgia, USA: Cooperative for Assistance and Relief Everywhere; 2008. Available: https://www.researchgate.net/publica tion/259999318_The_Coping_Strategies_Index_Field_Methods_Manual_-_Second_Edition. Accessed: 23 September 2024.

37 Jomaa L, Naja F, Cheaib R, Hwalla N. Household food insecurity is associated with a higher burden of obesity and risk of dietary inadequacies among mothers in Beirut, Lebanon. BMC Public Health. 2017;17(1):567. Medline:28606120 doi:10.1186/s12889-017-4317-5

38 World Food Programme Vulnerability Analysis and Mapping Branch. Food Consumption Analysis: Calculation and use of the food consumption score in food security analysis. Rome, Italy: World Food Programme Vulnerability Analysis and Mapping Branch; 2008. Available: https://documents.wfp.org/stellent/groups/public/documents/manual_guide_proced/wfp197216.pdf. Accessed: 23 September 2024.

39 Ballard T, Coates J, Swindale A, Deitchler M. Household Hunger Scale: Indicator Definition and Measurement Guide. Washington, DC, USA: FHI 360; 2011. Available: https://www.fantaproject.org/sites/default/files/resources/HHS-Indicator-Guide-Aug2011.pdf. Accessed: 23 September 2024.

40 People In Need, IndiKit. Treatment of Drinking Water. Available: https://www.indikit.net/indicator/2-wash/59-treatment-of-drinking-water. Accessed: 24 September 2024.

41 People In Need, IndiKit. Use of Improved Sanitation Facility. Available: https://www.indikit.net/indicator/75-use-of-improved-sanitation-facility. Accessed: 24 September 2024.

42 People In Need, IndiKit. Disposal of Child Faeces. Available: https://www.indikit.net/indicator/77-disposal-of-child-faeces. Accessed: 24 September 2024.

43 Morakinyo OM, Fagbamibe AF, Adebowale AS. Decomposition of factors associated with housing material inequality in under-five deaths in low and middle-income countries. Archives of Public Health. 2022;80(1):13. Medline:34983645 doi:10.1186/s13690-021-00768-0

44 Miljko L. Exploratory Factor Analysis (EFA) - How to interpret KMO and Bartlett's test. Statistische Beratung; 2020. Available: https://statistical.agency/images/Exploratory_Factor_Analysis-EFA-How_to_interpret_KMO_and_Bartletts_test.pdf. Accessed: 23 September 2024.

45 Venugopal V, Dongre AR, Ponnusamy P. Constructing practical and realistic asset-based socioeconomic status assessment scale using principal component analysis for urban population of Puducherry, India. Indian J Community Med. 2021;46(3):494-98. Medline:34759495 doi:10.4103/ijcm.IJCM_925_20

46 Save the Children International, International Rescue Committee, Mercy Corps. Multipurpose Outcome Indicators and Guidance. London, UK: International Rescue Committee, Mercy Corps, Save the Children International; 2022. Available: https://resourcecentre.savethechildren.net/pdf/Grand-Bargain-MPC-Outcome-Indicators-Guidance.pdf/. Accessed: 23 September 2024.

47 Save the Children International, International Rescue Committee, Mercy Corps. Multipurpose Cash Assistance Monitoring & Evaluation: Baseline - Endline Analysis Guidance Note. London, UK: International Rescue Committee, Mercy Corps, Save the Children International. Available: https://resourcecentre.savethechildren.net/pdf/MPCA-Baseline-Endline-Analytical-Guidance-Note.pdf/. Accessed: 23 September 2024.

48 World Health Organization. Breastfeeding. Available: https://www.who.int/health-topics/breastfeeding#tab=tab_1. Accessed: 30 September 2024.

49 World Health Organization. Infant and young child feeding. 2023. Available: https://www.who.int/news-room/fact-sheets/detail/infant-and-young-child-feeding#:~:text=WHO%20provides%20simple,%20coherent%20and%20feasible%20guidance%20to%20countries%20for. Accessed: 30 September 2024.

50 People In Need, IndiKit. Hand Washing Knowledge. Available: https://www.indikit.net/indicator/2-wash/67-hand-washing-knowledge. Accessed: 25 September 2-24.

51 Jalata DD, Asefa BG. Trends and Determinants of Dietary Diversity in Children Aged 6–59 Months in Ethiopia: Analysis of 2005–2016 Demographic and Health Survey. Curr Dev Nutr. 2022;6(10):nzac135. Medline:36475020 doi:10.1093/cdn/nzac135

52 Diop L, Becquey E, Turowska Z, Huybregts L, Marie T R, Gelli A. Standard Minimum Dietary Diversity Indicators for Women or Infants and Young Children Are Good Predictors of Adequate Micronutrient Intakes in 24–59-Month-Old Children and Their Nonpregnant Nonbreastfeeding Mothers in Rural Burkina Faso. J Nutr. 2021;151(2):412–22. Medline:33326567 doi:10.1093/jn/nxaa360

53 Castet A. The impact of large-scale land acquisitions on child food insecurity in Africa. World Dev. 2024;179:106597. https://doi.org/10.1016/j.worlddev.2024.106597

54 United Nations Children's Fund. UNICEF Conceptual Framework on Maternal and Child Nutrition. New York, USA: United Nations Children's Fund; 2021. Available: https://www.unicef.org/media/113291/file/UNICEF%20Conceptual%20Framework.pdf. Accessed: 23 September 2024.

55 United Nations Children's Fund. Improving child nutrition: The achievable imperative for global progress. New York, NY, USA: United Nations Children's Fund; 2013. Available: https://data.unicef.org/resources/improving-child-nutrition-the-achievable-imperative-for-global-progress/. Accessed: 23 September 2024.

56 Victora CG, Huttly SR, Fuchs SC, Olinto MT. The role of conceptual frameworks in epidemiological analysis: a hierarchical approach. Int J Epidemiol. 1997;26(1):224–7. Medline:9126524 doi:10.1093/ije/26.1.224

57 Akseer N, Bhatti Z, Mashal T, Soofi S, Moineddin R, Black RE, et al. Geospatial inequalities and determinants of nutritional status among women and children in Afghanistan: an observational study. Lancet Glob Health. 2018;6(4):e447–59. Medline:29454554 doi:10.1016/S2214-109X(18)30025-1

58 Johnston R, Jones K, Manley D. Confounding and collinearity in regression analysis: a cautionary tale and an alternative procedure, illustrated by studies of British voting behaviour. Qual Quant. 2018;52(4):1957–76. Medline:29937587 doi:10.1007/s11135-017-0584-6
